# Nucleos(t)ide Analog Treatment Discontinuation in Chronic Hepatitis B Virus Infection: A Systematic Literature Review

**DOI:** 10.1016/j.gastha.2024.08.015

**Published:** 2024-08-23

**Authors:** Robert Gish, Kosh Agarwal, Anadi Mahajan, Supriya Desai, Saifuddin Kharawala, Rob Elston, Joyeta Das, Stuart Kendrick, Vera Gielen

**Affiliations:** 1 Hepatitis B Foundation, Doylestown, Pennsylvania; 2 Institute of Liver Studies, King’s College Hospital, London, UK; 3 Bridge Medical Consulting, London, UK; 4 Clinical Research, GSK, Stevenage, Hertfordshire, UK; 5 Hepatology Global Medical Affairs, GSK, London, UK; 6 Global Medical Affairs, GSK, Stevenage, Hertfordshire, UK; 7 Value Evidence and Outcomes, GSK, London, UK

**Keywords:** Antiviral Treatment, Evidence-Based Care, Viral Hepatitis B.

## Abstract

**Background and Aims:**

The aim of this systematic literature review (SLR) was to examine outcomes and associated predictors following nucleos(t)ide analog (NA) treatment cessation in adult patients with chronic hepatitis B virus infection.

**Methods:**

The SLR was conducted according to PRISMA methodology. All included studies were quality assessed using appropriate scales or checklists.

**Results:**

The SLR identified 145 studies. Cumulative rates of clinical relapse (40 studies), virological relapse (53 studies), biochemical relapse (10 studies) and retreatment events (14 studies) post NA cessation varied widely across studies (clinical relapse: 40%–65%, virological relapse: 75%–94%, biochemical relapse: 63%–73%, retreatment rates: 30%–78% at 24 and 144 weeks, respectively). Significant predictors with adequate evidence of clinical relapse included older age, male gender, and higher hepatitis B surface antigen (HBsAg) and hepatitis B virus DNA at baseline and end of treatment. HBsAg loss was reported in 25 studies, with overall median HBsAg loss rates ranging from 2% at 24 weeks (5 studies) to 11% at 192 weeks (2 studies) post NA cessation. There was adequate evidence for lower HBsAg level at baseline and end of treatment as a significant and consistent predictor of HBsAg loss.

**Conclusion:**

There is considerable heterogeneity among studies of NA cessation. Data are currently incomplete to provide strong recommendations for NA cessation or to identify patients who may benefit most from this approach in clinical practice. Further studies are required to provide clearer guidelines, and tools to assess and monitor patients who may benefit from NA treatment cessation.

## Introduction

Hepatitis B virus (HBV) infection is a major global health issue, with an estimated all-age prevalence in 2022 of 257.5 million people and 820,000 deaths from HBV-related diseases,^1^^,^^2^ and is associated with stigma, discrimination and poor health-related quality of life.^3^ Treatment of chronic HBV infection aims to prevent disease progression and reduce the risk of cirrhosis, liver transplant, hepatocellular carcinoma and death, by suppressing or eliminating HBV.^4^, ^5^, ^6^, ^7^ Other goals of treatment include decreasing infectivity, reducing stigma and discrimination, managing extrahepatic disease, improving quality of life and possibly preventing other cancers linked to HBV infection.^4^^,^^7^ Inclusion of the patient voice in treatment decisions is also emerging as a reason to treat.^8^ The ideal outcome for HBV therapy and recently endorsed as the elevated endpoint for new therapies is functional cure, defined as long-term (≥6 months) sustained hepatitis B surface antigen (HBsAg) and HBV DNA loss, with or without seroconversion to anti–hepatitis B surface antibody after cessation of all treatment.^9^^,^^10^

Nucleos(t)ide analogs (NAs), a first-line therapy for chronic HBV infection, effectively suppress HBV DNA with very few side effects. However, NA therapy rarely leads to clearance of HBsAg and chronic long-term treatment may be required.^4^, ^5^, ^6^ The need for chronic NA therapy was questioned when a subset of patients in a cessation study, cleared HBsAg after stopping NA therapy,^11^ leading to the hypothesis that NA cessation may promote HBsAg loss.^12^ While the mechanism of action for HBsAg clearance following NA cessation remains to be fully elucidated, it is clear that cessation allows HBV replication to take place and, in some cases, it is possible that this may stimulate the immune system to clear HBsAg; however, viral rebound often leads to clinical relapse.^12^ Furthermore, outcomes across NA cessation studies vary, and evidence regarding this strategy has been conflicting.^12^

There is a variety of perspectives for NA withdrawal. As noted, withdrawal may promote greater HBsAg clearance, in a subset of patients, and reduce medication costs.^12^ Potential negative consequences of stopping NAs largely reflect concerns around patient safety, including the risk of acute-on-chronic liver failure, the potential for fulminant or unremitting necroinflammation and the unpredictability of acute hepatitis flares; these events may lead to liver transplant or death in some patients, though data on safety outcomes are limited and highly heterogenous.^12^ Further possible negatives relating to NA cessation include the need for increased clinical monitoring and patient anxiety about HBV reactivation.^12^ Therefore, the risks of NA cessation must be balanced against the benefits.

Guidelines from the American Association for the Study of the Liver Diseases (AASLD), the European Association for the Study of the Liver (EASL), and the Asian Pacific Association for the Study of Liver (APASL) vary in their recommendations for NA cessation.^13^ AASLD and EASL guidelines agree that therapy can be stopped if HBsAg loss is achieved and persists for 1 year, while APASL recommend NA cessation 6 months after completion of immunosuppressive therapy in HBsAg-negative and anti–HBc-positive patients and in HBsAg-positive patients who had low baseline HBV DNA (<2000 IU/mL) and no advanced liver fibrosis or cirrhosis.^4^^,^^5^^,^^14^ However, such clearance is uncommon and instead the duration of therapy is generally based on hepatitis B e-antigen (HBeAg) status, duration of HBV DNA suppression, and presence of cirrhosis or decompensation.^5^ AASLD and EASL guidelines recommend indefinite antiviral therapy for patients with cirrhosis who are HBeAg-positive, while APASL guidelines do not provide any specific recommendation in these patients.^4^^,^^5^^,^^14^ For patients who are HBeAg-positive and noncirrhotic and have achieved stable seroconversion with undetectable HBV DNA, EASL and AASLD agree that NA therapy can be discontinued following 12 months of consolidation therapy.^4^^,^^5^ The APASL 2015 guidelines recommended cessation following 6 months in these patients, while no specific recommendation is made in the updated 2021 guidelines for these patients.^6^^,^^14^ For patients who are HBeAg negative and noncirrhotic, AASLD guidelines suggest that NA cessation may be considered in patients with HBsAg loss but note that there is insufficient evidence to definitively guide treatment decisions in these patients.^5^ EASL guidelines state that if virological suppression has been achieved for at least 3 years, discontinuation may be considered if close monitoring can be guaranteed.^4^

Given the lack of clarity around NA cessation, the main objective of this systematic literature review (SLR) was to examine the evidence on outcomes reported after NA treatment cessation and predictor variables associated with those outcomes, in patients with chronic HBV infection.

## Methods

### Identification of Relevant Studies

This SLR was conducted according to PRISMA methodology (Figure). Searches were completed in 2 stages (Table A1). Initial structured searches from inception to February 2020 were undertaken in Medical Literature Analysis and Retrieval System Online and Excerpta Medica Database (Embase), via Embase. Searches used Emtree terms, using index terms where available as well as titles and abstracts. Subsequent updated structured searches for additional studies of interest were undertaken in Medical Literature Analysis and Retrieval System Online and Embase from January 2020 to July 2022, via Embase. The searches overlapped for 1 month to ensure relevant articles were not missed. The search was supplemented by gray literature searches and back-referencing of primary studies and review articles. Relevant conference abstracts between 2020 and 2022 were hand searched to retrieve the latest clinical studies (APASL, AASLD, EASL).

**Figure:**
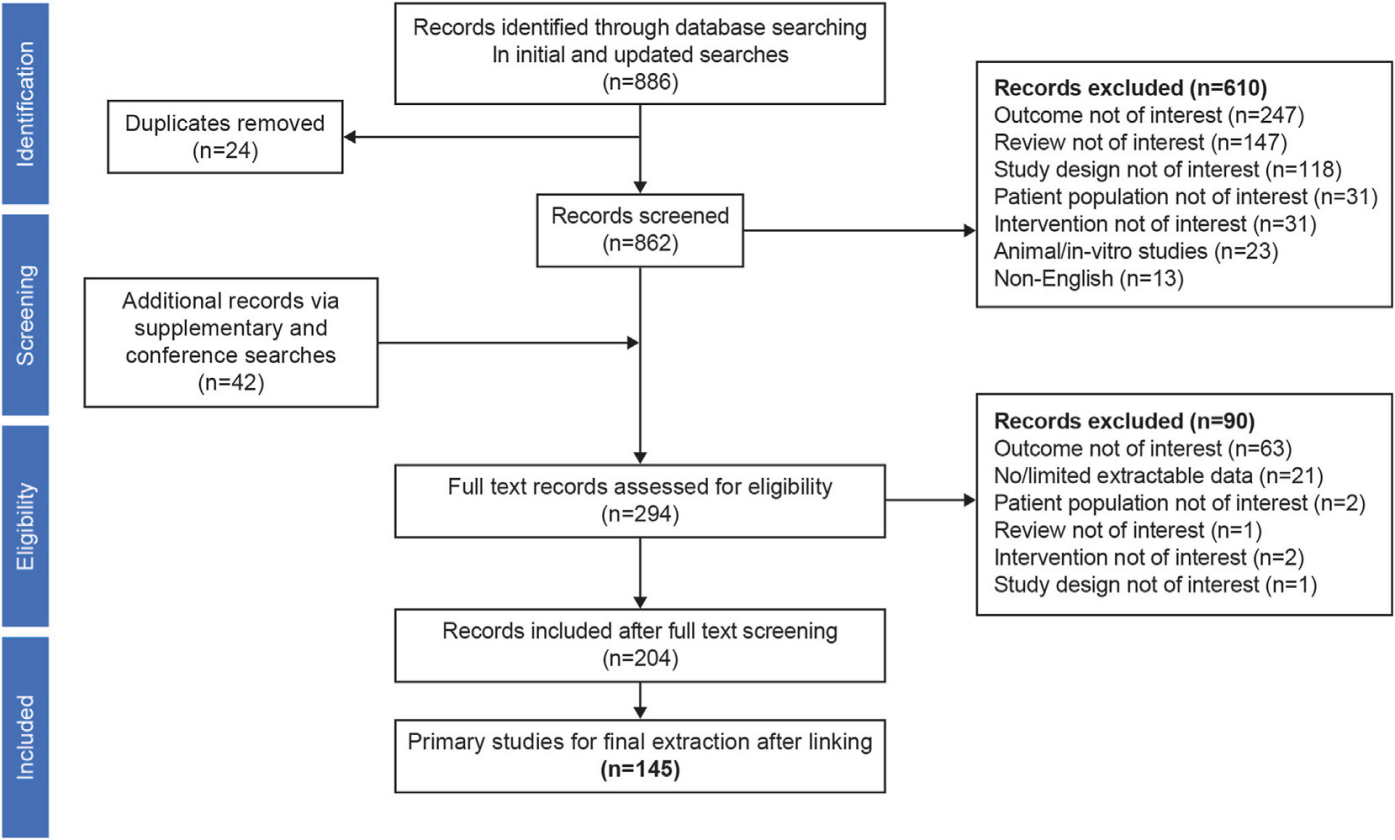
PRISMA Flow Diagram. Literature database searches for the updated searches (January 2020–July 2022) yielded 120 records. After removal of duplicates (n = 8), title and abstract screening of the remaining 112 citations was conducted, and 38 potentially relevant records were identified. To these, 36 citations identified through conference searches were added. Following a detailed examination of the full-text copies of these records, 41 unique studies (73 publications) met the inclusion criteria and were eligible for extraction.

### Study Selection

The title and abstract of each record were assessed for relevance before full-text versions were examined in more detail to determine the final list for inclusion. Studies were screened according to predefined criteria to include observational studies (prospective and retrospective cohort studies) and clinical trials in representative population of patients with chronic HBV infection who had discontinued therapy with any NA (Table A2). Each publication was assessed for inclusion by 2 independent reviewers and any discrepancies were resolved by a third reviewer.

### Data Collection and Extraction

Data extraction was performed by 1 researcher and checked by a second researcher. Outcomes of interest (categorical) reported after NA cessation included virological relapse (increase in HBV DNA), biochemical relapse (increase in alanine aminotransferase [ALT]), clinical relapse (increase in both HBV DNA and ALT), retreatment after discontinuation, HbsAg-related outcomes, HBeAg-related outcomes, and other clinical outcomes, for example, hepatic decompensation (HD), hepatic failure (HF), or hepatic-related death (HRD). Predictor variables associated with the given cessation outcomes of interest, such as demographic, clinical characteristics, and type of NA were also evaluated.

### Quality Assessment

Quality assessment for all included studies were completed using the appropriate scales or checklists: the Newcastle-Ottawa Scale for cohort studies, the National Institute for Health and Care Excellence checklist for randomized controlled trials (RCTs), and the Risk Of Bias In Nonrandomized Studies of Interventions checklist for non-RCTs.

## Results

### Search Results and Study Selection

Literature database searches yielded 886 citations, of which 145 unique studies (204 publications) were selected for final extraction and reporting (Figure). Most (92%, n = 133) were observational studies, of which 63 (43%) were retrospective cohort, 59 (41%) were prospective cohort, 11 (8%) were retrospective-prospective cohort studies, and the remainder (8%, n = 12) were clinical trials, including 9 (6%) RCTs and 3 (2%) non-RCTs (Figure A1). Two-thirds were journal articles (n = 96) and the rest (n = 49) were conference abstracts. Approximately 80% (n = 115) were published in the last 10 years (2013–2022). Data collection time points ranged from 1996 to 2021, although the period was not reported in 54 studies.

There was considerable heterogeneity among the studies. For example, mean follow-up ranged from 26.4 ^15^ to 385.8 weeks,^16^ and was not reported in 16 studies. The majority of data came from single-center studies (46%), multicenter studies (40%), and multicenter international studies (7%). Most (72%) of the single-center studies were conducted in Asian countries. Sample sizes ranged from 7 to 10,192 participants. A full list of the selected studies is available in Table A3.

### Quality Assessment of Studies

Approximately 83% of the observational studies were rated as fair to good quality. Of the 9 RCTs included in the SLR, 3 had a low risk of bias and for 6 the risk of bias was unclear (Table A4). The 3 non-RCTs had low risk of bias.

### Patient Characteristics in the Studies

Approximately two-fifths (n = 59) of studies reported patient age (median 28–63 years) and 35%–100% of participants were male in the 100 studies that reported gender (Table A5). ALT levels were reported using a variety of units across studies, with median values of up to 44.0 IU/L (Table A5). At the start of NA therapy, 27.8% to 98.1% of participants across 31 studies were HBeAg negative. A similar proportion (33.1%–100%) were HBeAg negative at NA cessation (n = 6 studies). HBeAg and HBV DNA levels were reported with IU/mL, IU/L log IU/mL, log U/mL, kU/mL, log copies/mL, μmol/L and mEq/mL. Median duration of NA treatment was 24 to 571.6 weeks (46 studies), and in 5 studies the median duration of total NA treatment was 148 to 160 weeks.

Nearly 65% (n = 94) studies included participants who had discontinued any NA therapy and 72% (n = 105) included those who had discontinued specific NA therapies, including lamivudine (n = 37); entecavir (ETV, n = 34); tenofovir disoproxil fumarate (TDF, n = 18); adefovir dipivoxil (n = 7); telbivudine (n = 7) and adefovir dipivoxil + lamivudine (n = 2).

### NA Therapy Cessation Criteria and Guidelines

The NA cessation criteria were specified in 62% (n = 90) studies and 38% (n = 55) studies did not clearly report any criteria (Table 1). Of the 12 RCTs, 5 listed NA cessation criteria while 7 did not. Nearly 64% (85 of 135) of the observational studies listed NA cessation criteria. NA cessation criteria reflected the local guidelines and clinical practice as well as patients’ decision and treating physicians’ judgment.

**Table 1:** NA Therapy Cessation Criteria and Stopping Rules Used Across Selected Studies

	Number of studies, n (%)
Overall (N = 145)	
Not clearly reported	55 (38)
Criteria or stopping rules specified	90 (62)
Clinical trials (N = 12)	
Not clearly reported	7 (58)
Per clinical criteria	5 (42)
Observational studies (N = 133)^a^	
Not clearly reported	48 (36)
In accordance with international clinical practice guidelines	60 (45)
APASL	51 (84)
AASLD	8 (13)
Taiwan’s NHI Plan or Policy	4 (3)
EASL	3 (2)
CMA	2 (2)
Chinese Hepatitis B management	1 (1)
Asian-Pacific Consensus statement on the management of chronic hepatitis B	1 (1)
KASL	1 (1)
Per clinical or other criteria	24 (18)
Based on physician advice or patient decision or both	8 (6)
Based on serological response criteria for HBV DNA, ALT, HBeAg or HBsAg without consolidation treatment	8 (6)
Based on serological response criteria for HBV DNA, ALT, HBeAg or HBsAg with consolidation treatment	6 (5)
Owing to economic burden of long-term NA therapy	2 (2)
Based on multiple NA stopping criteria (clinical or other)	1 (1)
Based on the duration of treatment	1 (1)
Cited use of both guidelines and other or clinical criteria	1 (1)

AASLD, American Association for the Study of Liver Diseases; ALT, alanine aminotransferase; APASL, Asian-Pacific Association for the Study of the Liver; CMA, Chinese Medical Association; EASL, European Association for the Study of the Liver; HBeAg, hepatitis B e-antigen; HBsAg, hepatitis B surface antigen; HBV, hepatitis B virus; HBV DNA, hepatitis B virus deoxyribonucleic acid; KASL, Korean Association for the Study of the Liver; NA, nucleos(t)ide analogue; NHI, National Health Insurance; PRISMA, Preferred Reporting Items for Systematic reviews and Meta-Analyses.

a Total may exceed N, as some studies reported use of more than one guideline or criterion.

Of the observational studies who reported the cessation criteria (n = 85), most followed clinical practice guidelines (n = 60) particularly the APASL (n = 51) and AASLD (n = 8). Twenty-four studies reported clinical criteria such as physician judgment, serological response, economic factors related to NA cost (e.g. Taiwan government policy), multiple criteria, duration of treatment and a combination of guidelines and clinical criteria (Table 1).

### Clinical Relapse Rates Post NA Cessation

Clinical relapse rates were reported in 40 studies (all observational). Most (70% [n = 28]) studies defined clinical relapse as “cumulative incidence of serum HBV DNA ≥2000 international unit (IU)/mL and serum ALT ≥2× upper limit of normal (ULN)”, while 18% (n = 7) of studies did not report any definition (Table A6), and the remaining studies used varying definitions (Table A7). Cumulative clinical relapse rates varied depending on the definition of clinical relapse used in the study and varied from 40% at 24 weeks to 65% at 144 weeks. In most studies, the majority of clinical relapse events (≥60%) had occurred by 96 to 144 weeks post NA cessation (Table 2). Higher clinical relapse rates were reported in patients who had received TDF vs other specific NA therapies at most key time points. Clinical relapse rates were lower in HBeAg-negative patents compared with HBeAg-positive patients at most key time points in 2 of the studies.

**Table 2:** Cumulative Clinical Relapse Rates Following NA Cessation

Clinical relapse rates (range, median^a^) at key timepoints post NA cessation							
Study group	No. of studies	24 wk	48 wk	96 wk	144 wk	192 wk	240 wk
Observational studies							
NA-D (any)	21	0.0%–40.0%, median: 24.82 (n = 8)	0.0%–53.3%, median: 32.25 (n = 19)	0.0%–60.0%, median: 35 (n = 13)	0.0%–65.0%, median: 38.82 (n = 11)	34.4%–53.1% (n = 2)	-
ADV-D	1	12.6% (n = 1)	-	12.6% (n = 1)	-	-	-
ETV-D	17	0.0%–46.8%, median: 10.2 (n = 12)	20.0%–72.7%, median: 30 (n = 15)	17.39%–81.0%, median: 46 (n = 11)	2.4%–55.5%, median: 47.25 (n = 5)	60.0% (n = 1)	-
LAM-D	3	20.8%–100.0% (n = 2)	27.1%–35.1% (n = 1)	20.0%–57.5% (n = 2)	-	-	
LdT-D	3	2.6%–7.7% (n = 2)	0.0%–40.0% (n = 2)	0.0%–46.8% (n = 2)	0.0%–46.8% (n = 1)	23.3% (n = 1)	-
TDF-D	13	27.0%–63.4%, median: 34.2 (n = 10)	7.2%–100.0%, median: 45.95 (n = 12)	10.8%–95.4%, median: 62.4 (n = 8)	10.3%–76.0%, median: 60 (n = 5)	60.0% (n = 1)	-
ADV + LAM-D	1	40.7% (n = 1)	-	50.8% (n = 1)	-	-	-
HBeAg-(NA start)	12	6.8%–59.2% (n = 9)	23.0%–43.5% (n = 12)	23.3%–56.1% (n = 11)	6.7%–61.8% (n = 5)	53.0% (n = 1)	-
HBeAg+(NA start)	11	5.4%–63.4% (n = 9)	22.5%–57.9% (n = 10)	8.9%–73.7% (n = 10)	1.8%–68.5% (n = 4)	48.0% (n = 1)	-

ADV, adefovir disoproxil; -D, discontinued; ETV, entecavir; HBeAg-, hepatitis B e-antigen negative; HBeAg+, hepatitis B e-antigen positive; LAM, lamivudine; LdT, telbivudine; n, number of studies; TDF, tenofovir disoproxil fumarate.

a Median values are not provided if ≤ 2 studies contributed to the outcome.

### Virological and Biochemical Relapse Rates Post NA Cessation

Data were reported in 53 studies (all observational studies) for virological relapse and 10 studies (9 observational studies and 1 clinical trial) for biochemical relapse. Most (55% [n = 29]) defined virological relapse as “cumulative incidence of serum HBV DNA ≥2000 IU/mL in either a single or 2 distinct measurements, regardless of ALT level, 9% (n = 5) did not report a virological relapse definition and the remaining studies used varying definitions (Table A7). The cumulative rate of virological relapse varied widely in relation to the definition of virological relapse followed across studies from 75% at 24 weeks to 94% at 144 weeks. Similarly for biochemical relapse, a range of definitions were used for reporting cumulative incidence (e.g. ALT >2x ULN [n = 3], ALT ≥5x ULN [n = 2], ALT >10x ULN [n = 2], ALT ≥5x or 10x ULN [n = 1], ALT > ULN [n = 1], per specific cut-off values for ALT of >50 to >500 IU/mL [n = 3]). Rates of biochemical relapse ranged from 63% at 24 weeks to 73% at 144 weeks. Most virological relapse (≥90%) and biochemical relapse (>70%) events, irrespective of definition, had occurred by 48 to 96 weeks post NA cessation (Tables A8 and A9).

### Retreatment Rates Post NA Cessation

Data on retreatment rates post NA cessation were reported in 14 studies (all observational). Four (29%) studies defined the criteria for retreatment after NA cessation in line with Taiwan’s National Health Insurance Plan. Retreatment rates varied from 30% at 24 weeks to 78% at 144 weeks. Most retreatment events (≥70%) had occurred by 96 to 144 weeks post NA cessation (Table A10).

### HBsAg- and HBeAg-Related Outcomes Post NA Cessation

HBsAg loss was reported for 25 studies (24 observational studies and 1 RCT). Three studies (12%) defined HBsAg loss in terms of functional cure or undetectable HBsAg at any time during off-treatment follow-up. HBsAg seroconversion and HBsAg seroreversion were reported in 1 study each. Varying HBsAg loss was observed, with overall median HBsAg loss rates ranging from 2% at 24 weeks (n = 5 studies) to 11% at 192 weeks (n = 2 studies) post NA cessation (Table 3). Following cessation of TDF, median HBsAg loss rates ranged from 2% at 24 weeks (n = 1 study) to 15% at 144 weeks (n = 4 studies). Following cessation of ETV, rates ranged from 1% at 24 weeks (n = 1 study) to 10% at 144 weeks (n = 3 studies). HBeAg-related outcomes were reported for 9 studies (all observational) including HBeAg loss (n = 1), HBeAg seroconversion (n = 1), and HBeAg seroreversion (n = 7). HBeAg seroreversion rates ranged from 0% at 24 weeks (n = 1 study) to 12% at 144 and 192 weeks (n = 1 study) (Table A11).

**Table 3:** Cumulative Rates for HBsAg-Related Outcomes

HBsAg loss rates (range, median^a^) at key timepoints post NA cessation							
Study group	No. of studies	24 wk	48 wk	96 wk	144 wk	192 wk	240 wk
Clinical trials—HBsAg loss							
NA-D (any)	1	-	1.1%^b^ (n = 1)	-	-	-	-
Observational studies—HBsAg loss							
NA-D (any)	19	0.0%–63.0%, median: 2 (n = 5)	0.0%–99.5%, median: 3.33 (n = 13)	0.0%–75.2%, median: 7.21 (n = 9)	0.0%–79.0%, median: 10.29 (n = 11)	0.0%–79.5%	-
ETV-D	3	0.0%–1.1% (n = 1)	1.0%–2.1% (n = 1)	3.0%–7.0% (n = 2)	4.5%–29.4%, median: 9.6 (n = 3)	10.0% (n = 1)	-
TDF-D	5	1.1%–2.9% (n = 1)	4.0%–15.5% (n = 2)	0.0%–59.3%, median: 9.1 (n = 4)	8.0%–42.5%, median: 14.5 (n = 4)	13.9% (n = 1)	-
HBeAg-(NA start)	6	1.0%–5.0% (n = 2)	1.8%–16.0%, median: 2.55 (n = 3)	8.5% (n = 1)	10.0%–15.4%, median: 12.6 (n = 4)	12.1% (n = 1)	-
HBeAg+(NA start)	4	1.0% (n = 1)	3.0% (n = 1)	0.0%–9.4% (n = 2)	5.6%–9.4%, median: 9 (n = 3)	9.0% (n = 1)	-
Observational studies—HBsAg seroconversion							
NA-D (any)	1	-	-	-	60.9% (n = 1)	-	-
Observational studies—HBsAg seroreversion							
NA-D (any)	1	-	0.8% (n = 1)	-	2.3% (n = 1)	-	-

ADV, adefovir disoproxil; -D, discontinued; HBeAg-, hepatitis B e-antigen negative; HBeAg+, hepatitis B e-antigen positive; LAM, lamivudine; LdT, telbivudine; n, number of studies; NA, nucleos(t)ide analogue; TDF, tenofovir disoproxil fumarate.

a Median values are not provided if ≤ 2 studies contributed to the outcome.

b 3.3% at 120 wk in the same clinical trial (n = 1).

### Other Clinical Outcomes

Data were reported in 8 studies (all observational) for other clinical outcomes including hazard ratio (n = 6),^16^, ^17^, ^18^, ^19^, ^20^, ^21^ HF (n = 1) and HRD (n = 3). The number of patients included in studies reporting HD ranged from 144^20^ to 8631;^19^ duration of follow-up varied from less than 52 weeks^21^ to 385.5 weeks. ^16^ HD rates ranged from 0% to 17% at 24 weeks, 0% to 23% at 48 weeks, 1% to 15% at 96 weeks, and 1% to 17% at 144 weeks (Table A12).

### Predictors of Outcomes

Predictors of virological relapse were the most reported (n = 43 studies), followed by predictors of clinical relapse (n = 32 studies) and predictors of HBsAg loss (n = 18 studies). Few studies reported predictors of biochemical relapse (n = 7), retreatment after NA cessation (n = 6) and HD (n = 5). Predictors of HBeAg seroconversion, HBeAg seroreversion and HF were reported in 1 study each. Based on data from 6 studies, predictors of HD or HF included male gender, older age, higher bilirubin, higher composite ALT and Fib-4 scores, presence of liver fibrosis (higher Fib-4 end-of-treatment [EOT] scores), presence of liver cirrhosis, prior HD or HF, and tenofovir (vs ETV) as type of discontinued NA therapy.^12^^,^^16^^,^^19^^,^^20^^,^^22^^,^^23^ No studies reported data for predictors of HBsAg seroconversion, HBsAg seroreversion, and HRD. Significant predictors of clinical relapse with adequate evidence included higher HBsAg and HBV DNA at baseline and EOT, older age, and male gender (Table 4). There was limited evidence that the risk of clinical relapse is increased following cessation of TDF vs that of ETV (Table 4). Significant predictors of virological relapse with adequate evidence included higher levels of certain viral markers (HBsAg, hepatitis B core-related antigen [HBcrAg] and HBV DNA at baseline and EOT and HBV RNA at EOT), older age, and shorter duration of NA consolidation (Table A13). Higher HBV RNA serum levels were associated with a higher risk of virological relapse (n = 5 studies),^24^, ^25^, ^26^, ^27^, ^28^ clinical relapse (n = 3 studies),^25^^,^^29^^,^^30^ biochemical relapse (n = 2 studies),^31^^,^^32^ and retreatment after discontinuation (n = 1 study).^28^ In terms of predictors of HBsAg loss, there was adequate evidence for lower HBsAg level at baseline and EOT as a significant and consistent predictor (Table 5).

**Table 4:** Summary of Significant Predictors of Clinical Relapse Following NA Cessation

Variable or factor	Available literature	Consistency of association	Summary finding (based on quantitative multivariate analysis)
Demographic variables			
Age	Adequate	Consistent	• ↑ age a/w ↑ risk of clinical relapse (HR: 1.029–2.120) (n = 7 studies) • ↑ age a/w ↑ risk of clinical relapse (OR: 3.060–6.050, 95% CI: NR) (n = 1 study)
Gender	Adequate	Consistent	• Male gender a/w ↑ risk of clinical relapse (vs female gender) (HR: 1.600–7.560) (n = 5 studies) • Male gender a/w ↑ risk of clinical relapse (vs female gender) (OR: 9.810, 95% CI: NR) (n = 1 study)
Biochemical variables			
ALT	Limited	Inconsistent	• ↑ ALT (at NA start) a/w ↑ risk of clinical relapse (HR: 1.400, 95% CI: NR) (n = 1 study) • ↑ ALT (at NA start) a/w ↓ risk of clinical relapse (HR: 0.990–0.996) (n = 2 studies)
Bilirubin	Very limited	Consistent	• ↑ total bilirubin ≥2 mg/dL (at NA start) a/w ↓ risk of clinical relapse (HR: 0.676, 95% CI: 0.475–0.962) (n = 1 study)
Viral markers			
HBsAg	Adequate	Consistent	• ↑ HBsAg (BL/EOT) a/w ↑ risk of clinical relapse (HR: 1.014–9.500) (n = 14 studies) • ↑ HBsAg (EOT) a/w ↑ risk of clinical relapse (OR: 1.500, 95% CI: 1.100–2.000) (n = 1 study) • ↑ HBsAg (EOT) > 200 IU/mL (vs ≤200 IU/mL) a/w ↑ risk of clinical relapse (HR: 3.573, 95% CI: 1.190–10.733) (n = 1 study) • ↑ HBsAg (EOT) > 200 IU/mL (vs <50 IU/mL) a/w ↑ risk of clinical relapse (HR: 9.070, 95% CI: 1.230–66.640) (n = 1 study) • ↑ qHBsAg (EOT) > 100 IU/mL (vs ≤100 IU/mL) a/w ↑ risk of clinical relapse (HR: 3.160, 95% CI: 1.250–7.890) (n = 1 study) • ↑ HBsAg (EOT) ≤80 IU/mL (vs >80 IU/mL) a/w ↓ risk of clinical relapse (HR: 0.230, 95% CI: 0.090–0.580) (n = 1 study) • ↑ HBsAg <2 log10 IU/mL (vs >2 log10 IU/mL) a/w ↓ risk of clinical relapse (OR: 6.686, 95% CI: 1.703–26.255) (n = 1 study)
HBV DNA	Adequate	Consistent	• ↑ HBV DNA (BL) a/w ↑ risk of clinical relapse (HR: 1.010–1.740) (n = 4 studies) • ↑ HBV DNA at 1-mo post EOT a/w ↑ risk of clinical relapse (HR: 1.510, 95% CI: 1.120–2.030) (n = 1 study) • ↑ HBV DNA (BL) a/w ↓ risk of clinical relapse (RR: 0.298, 95% CI: 0.128–0.688) (n = 1 study) • HBV DNA >4.7 x 10^3^ genomes/mL a/w ↑ risk of clinical relapse (OR: 1.947, 95% CI: 1.423–2.665) (n = 1 study) • HBV DNA >2 x 10^5^ IU/mL (BL) (vs <2 x 10^5^ IU/mL) in all patients (OR: 3.934, 95% CI: 1.345–11.508) and in noncirrhotic patients (OR: 14.500, 95% CI: 1.945–108.173) a/w ↑ risk of clinical relapse (n = 1 study)
HBV RNA	Limited	Consistent	• ↑ HBV RNA (EOT) a/w ↑ risk of clinical relapse (HR: 1.320, 95% CI: 1.020–1.700) (n = 1 study) • HBV RNA positivity a/w ↑ risk of clinical relapse (HR: 3.580, 95% CI: 1.264–1.264) (n = 1 study) • HBV RNA positivity a/w ↑ risk of clinical relapse (OR: 3.453, 95% CI: 1.387–8.597) (n = 1 study)
HBcrAg	Limited	Consistent	• ↑ HBcrAg (EOT/BL) a/w ↑ risk of clinical relapse (HR: 1.922–2.210) (n = 2 studies) • ≥4 (vs <4 log10 U/mL) HBcrAg (EOT) a/w ↑ risk of clinical relapse (HR: 5.696, 95% CI: 1.371–23.670) (n = 1 study) • <4 (vs ≥4 log10 U/mL) HBcrAg (EOT) a/w ↑ risk of clinical relapse (OR: 3.702, 95% CI: 1.614–8.488) (n = 1 study)
HBV genotype	Limited	Inconsistent	• HBV genotype C (vs B) a/w ↑ risk of clinical relapse (HR: 2.960, 95% CI: 1.320–6.650) (n = 1 study) • HBV genotype C (vs B) a/w ↓ risk of clinical relapse (HR: 0.496, 95% CI: 0.330–0.746) (n = 1 study) • HBV genotype B (vs C) a/w ↑ risk of clinical relapse (HR: 2.10, 95% CI: 1.440–3.170) (n = 1 study)
HBV DNA + HBV RNA	Very limited	Consistent	• HBV DNA + HBV RNA positivity (EOT) a/w ↑ risk of clinical relapse (HR: 4.549, 95% CI: 1.089–19.002) (n = 1 study)
HBsAg + HBV DNA	Very limited	Consistent	• HBsAg ≥40 IU/mL (EOT) + HBV DNA of 5 x 10^4^ IU/mL (BL) a/w ↑ risk of clinical relapse (HR: 2.680, 95% CI: 1.830–3.930) (n = 1 study)
HBsAg + HBcrAg	Very limited	Consistent	• HBsAg ≥40 IU/mL (EOT) + HBcrAg of 4 log U/mL (BL) a/w ↑ risk of clinical relapse (3.020, 95% CI: 2.030–4.500) (n = 1 study)
Anti-HBc	Very limited	Consistent	• ↑ anti-HBc a/w ↓ risk of clinical relapse (HR: 0.310, 95% CI: 0.150–0.650) (n = 1 study)
NA treatment-related variables			
Duration of NA consolidation	Limited	Consistent	• Longer duration of NA consolidation a/w ↓ risk of clinical relapse (HR: 0.222–0.490) (n = 2 studies) • Longer duration of NA consolidation a/w ↓ risk of clinical relapse (OR: 0.99, 95% CI: 0.990–0.990) (n = 1 study)
Type of NA Rx-D	Limited	Consistent	• TDF therapy (vs ETV therapy) a/w ↑ risk of clinical relapse (HR: 1.425–2.690) (n = 4 studies)
Prior NA exp.	Limited	Consistent	• Prior NA experience a/w ↑ risk of clinical relapse (HR: 1.425–2.690) (n = 4 studies)
Other reported variables			
APASL Rx endpoint	Very limited	Consistent	• Achievement of APASL treatment endpoint a/w ↓ risk of clinical relapse (HR: 0.310, 95% CI: 0.150–0.650) (n = 1 study)
SAE, as initial indication for starting NA therapy	Very limited	Consistent	• SAE (hepatitis B flare; i.e., HBV viral load >2000 IU/mL and ALT >5x ULN with jaundice (total bilirubin ≥2 mg/dL), and/or coagulopathy (prolonged prothrombin time ≥3 s) as the initial indication for starting NA therapy (SAE group) compared with the non-SAE group a/w ↑ risk of clinical relapse (1.790, 95% CI: 1.040–3.060) (n = 1 study)
HBeAg loss	Very limited	Consistent	• HBeAg loss beyond 2 y during NA therapy (vs under 2 y) a/w ↓ risk of clinical relapse (OR: 0.15, 95% CI: NR) (n = 1 study)
Time to ALT normalization	Very limited	Consistent	• Time to ALT normalisation within 3 mo (vs >3 mo) a/w ↓ risk of clinical relapse (0.239, 95% CI: 0.088–0.648) (n = 1 study)
Duration of HBV DNA of <0.7 log IU/mL	Very limited	Consistent	• Duration of HBV DNA of <0.7 log IU/mL (mo) a/w ↓ risk of clinical relapse a/w (HR: 0.943, 95% CI: 0.898–0.990) (n = 1 study)
SCALE-B score	Very limited	Consistent	• SCALE-B score ≥320 (highest strata) vs 260 (lowest strata) a/w ↑ risk of clinical relapse (HR: 8.500, 95% CI: 1.100–64.300) (n = 1 study)

Available literature: very limited (1 study only); limited (2–4 studies); adequate (≥5 studies). SCALE-B, score = 35 ×HBsAg + 20 × HBcrAg + 2 × age + 40 for TDF use.

ALT, alanine aminotransferase; Anti-HBc, antibodies of hepatic B core antigen; APASL, The Asian-Pacific Association for the Study of the Liver; a/w, associated with; neg, negative; BL, baseline; CI, confidence interval; -D, discontinued; DNA, deoxynucleic acid; EOT, end of treatment; exp., experience; Ex(p) B, exponential B coefficient; HBV, hepatitis B virus; HBcrAg, hepatitis B core-related antigen; HBeAg, hepatitis B e-antigen; HR, hazard ratio; NA, nucleos(t)ide analogue; NR, not reported; OR, odds ratio; RNA, ribonucleic acid; RR, risk ratio; Rx, treatment; Rx-D, treatment discontinued; SAE, severe acute exacerbation; SC, seroconversion; TDF, tenofovir disoproxil fumarate; UD, undetectable; VR, virological relapse.

**Table 5:** Summary of Significant Predictors of HBsAg Loss Following NA Cessation

Variable or factor	Available literature	Consistency of association	Summary finding (based on quantitative multivariate analysis)
Demographic variables			
Age	Limited	Consistent	• ↑ age a/w ↓ likelihood of HBsAg loss (HR: 0.958–0.964) (n = 2 studies)
Gender	Very limited	Consistent	• Male gender a/w ↑ likelihood of HBsAg loss (vs Female gender) (OR: 1.900, 95% CI: 1.100–3.600) (n = 1 study)
Biochemical variables			
ALT	Very limited	Consistent	• ↑ ALT (at 1 mo of NA Rx) a/w ↑ likelihood of HBsAg loss (HR: 1.26, 95% CI: 1.08–1.47) (n = 1 study)
Platelet count	Very limited	Consistent	• ↑ Platelet count a/w ↑ likelihood of HBsAg loss (HR: 1.011, 95% CI: 1.000–1.022) (n = 1 study)
Viral markers			
HBsAg	Adequate	Consistent	• ↑ HBsAg (BL/EOT) a/w ↓ likelihood of HBsAg (HR: 0.152–0.731) (n = 12 studies) • ↑ on-treatment HBsAg log10 decline (BL to 1-mo) a/w ↑ HBsAg loss (HR: 42.28, 95% CI: 2.14–835.671) (n = 1 study) • ↑ HBsAg (EOT) a/w ↓ likelihood of HBsAg loss (OR: 0.380, 95% CI: NR) (n = 1 study) • ↑ HBsAg (BL) > 100 IU/mL (vs ≤100 IU/mL) a/w ↓ HBsAg loss (HR: 0.490, 95% CI: 0.120–0.199) (n = 1 study) • ↓ HBsAg (EOT) ≤100 IU/mL (vs 100–300 IU/mL, ≥300 IU/mL) a/w ↑ HBsAg loss (OR: 8.770–32.260) (n = 2 studies) • ↓ HBsAg (EOT) < 100 IU/mL (vs ≥100 IU/mL) a/w ↑ likelihood of HBsAg loss (HR: 22.500, 95% CI: 13.100–38.700) (n = 1 study) • ↑ on-treatment HBsAg decline a/w ↓ likelihood of HBsAg loss (OR: 0.100, 95% CI: 0.016–0.632) (n = 1) • ↓ HBsAg (EOT) a/w ↑ likelihood of HBsAg loss (HR: 2.240–9.850, 95% CI: 1.070–3.980, 4.570–24.390) (n = 1 study)^a^
HBV DNA	Limited	Consistent	• ↑ HBV DNA (BL) a/w ↓ likelihood of HBsAg loss (HR: 0.720, 95% CI: 0.590–0.890) (n = 1 study) • HBV DNA negativity (vs positivity) by ddPCR (BL) a/w ↑ likelihood of HBsAg loss (OR: 5.800, 95% CI: 1.300–23.600) (n = 1 study)
HBcrAg	Limited	Consistent	• ↑ HBcrAg (EOT) a/w ↓ likelihood of HBsAg loss (HR: 0.257–0.736) (n = 2 studies) • ↑ HBcrAg (EOT) a/w ↓ likelihood of HBsAg loss (OR: 0.476, 95% CI: NR) (n = 1 study)
HBV genotype	Limited	Inconsistent	• HBV genotype C (vs B) a/w ↑ likelihood of HBsAg loss (HR: 2.494–5.420) (n = 4 studies) • HBV genotype C (vs A) a/w ↓ likelihood of HBsAg loss (HR: 0.417, 95% CI: 0.137–1.265) (n = 1 study) • HBV genotype B (vs A) a/w ↓ likelihood of HBsAg loss (HR: 0.178, 95% CI: 0.060–0.532) (n = 1 study) • HBV genotype D (vs A) a/w ↑ likelihood of HBsAg loss (HR: 1.759, 95% CI: 0.514–6.014) (n = 1 study)
HBsAg + HBV DNA	Very limited	Consistent	• HBV DNA negativity by ddPCR (BL) + HBsAg <500 IU/mL (EOT) a/w ↑ HBsAg loss (HR: 15.8, 95% CI: 1.6–152.2) (n = 1 study)
NA treatment-related variables			
Duration of NA consolidation	Very limited	Consistent	• Longer duration of NA consolidation a/w ↑ likelihood of HBsAg loss (HR: 1.340, 95% CI: 1.020–1.750) (n = 1 study)
Duration of NA Rx.	Limited	Consistent	• Longer duration of NA Rx a/w ↑ likelihood of HBsAg loss (HR: 1.005–1.020) (n = 2 studies)
Retreatment	Very limited	Consistent	• Retreatment Yes (vs No) a/w ↓ likelihood of HBsAg loss (HR: 0.085, 95% CI: 0.018–0.39) (n = 1 study)
Other reported variables			
Virological relapse	Very limited	Consistent	• Noncirrhotic patients without VR (vs with VR) a/w ↑ likelihood of HBsAg loss (HR: 12.5, 95% CI: NR) (n = 1 study)
Time to UD HBV DNA	Very limited	Consistent	• Lower time to undetectable HBV DNA a/w ↑ HBsAg loss (HR: 2.270–3.040 per 2 analyses) (n = 1 study)
SCALE-B score	Limited	Consistent	• SCALE-B score ≥320 (highest strata) vs 260 (lowest strata) a/w ↓ HBsAg loss (HR: 0.040, 95% CI: 0.004–0.430) (n = 1 study) • SCALE-B score (per 1000 increase) a/w ↓ likelihood of HBsAg loss (HR: 0.930, 95% CI: 0.870–0.980) (n = 1 study)

Available literature: very limited (1 study only); limited (2–4 studies); adequate (≥5 studies). SCALE-B, score = 35 ×HBsAg + 20 × HBcrAg + 2 × age + 40 for TDF use.

a/w, associated with; ALT, alanine aminotransferase; BL, baseline; CI, confidence interval; CR, clinical relapse; -D, discontinued; ddPCR, droplet digital polymerase chain reaction; HBsAg, hepatitis B surface antigen; HBV, hepatitis B virus DNA, deoxynucleic acid; HBcrAg, hepatitis B core-related antigen; HR, hazard ratio; NA, nucleos(t)ide analogue; NR, not reported; OR, odds ratio; RNA, ribonucleic acid; Rx, treatment; UD, undetectable; VR, virological relapse.

a HR: Lower HBsAg level (categorical) was associated with higher likelihood of HBsAg loss in multiple analyses conducted in one study.^18^

## Discussion

This SLR evaluated the evidence on outcomes reported after NA treatment cessation and the predictor variables associated with those outcomes. Across all endpoints examined, the data were spread over a wide range, reflecting large within- and between-study variability. Many of the studies did not report specific definitions for post NA cessation outcomes and most of these were conference abstracts, especially for virological relapse and clinical relapse. Large variations were observed in outcomes between different studies that might be explained by the considerable heterogeneity across the studies included in this SLR in terms of study design, geography, follow-up duration, study population, outcome definitions, and the NA cessation criteria followed.

Cumulative rates of clinical relapse, virological relapse, biochemical relapse and retreatment events post NA cessation varied across studies, from 40% at 24 weeks to 65% at 144 weeks for clinical relapse, 75% at 24 weeks to 94% at 144 weeks for virological relapse, and 63% at 24 weeks to 73% at 144 weeks for biochemical relapse. Significant predictors with adequate evidence of clinical relapse included higher HBsAg and HBV DNA at NA initiation and EOT, older age, and male gender. Significant predictors with adequate evidence of virological relapse included higher levels of certain viral markers (HBsAg, HBcrAg and HBV DNA at baseline and EOT and HBV RNA at EOT), older age, and shorter duration of NA consolidation. However, it is worth noting that there was large between-study variability in study characteristics such as mean follow-up duration (38.5–385.8 weeks) and study setting (72% of the studies were conducted in Asia), along with a wide range of definitions used across studies (Table A7). Variations in follow-up duration are of particular relevance to cumulative relapse rates, as these increased over time following NA cessation, with some events occurring many months postcessation. In the RETRACT-B study, rates of all outcomes (HBsAg loss, virological relapse, biochemical relapse, retreatment) increased over time, indicating the importance of the length of follow-up in the interpretation of results.^33^ The minimum duration of follow-up was only 38.5 weeks,^34^ meaning that relapse events could have been missed over such a relatively short duration.

Data on outcome rates and predictors were limited for HD, HF, and HRD. HD rates were reported in 6 studies and ranged from 0% to 17% at 24 weeks, 0% to 23% at 48 weeks, 1% to 15% at 96 weeks, and 1% to 17% at 144 weeks. Additionally, only 1 study reported HF rates (0.4%), and HRD was reported in 3 studies with rates ranging from 0% to 0.3% at 48 weeks, 0.4% at 96 weeks, and 0.5% at 144 weeks.

The findings of this SLR confirm that NA withdrawal as a strategy to induce HBsAg loss yields mixed results^35^; however, HBsAg loss was only reported in 25 studies. Median HBsAg loss rates ranged from 0.6% at 24 weeks (range: 0%–63%) to 14% at 192 weeks (range: 0%–79.5%) post NA cessation, and were slightly higher with TDF vs ETV. There was adequate evidence for lower HBsAg level at baseline and EOT as a significant and consistent predictor for HBsAg loss but limited evidence for other predictors, such as race or genotype.

White racial heritage has been shown to be a predictor of outcomes in some studies^36^; however, given that most studies in this SLR were conducted in Asia, within-study comparative data are limited. It has been shown that Asian people often acquire HBV infection during early stages of life, through either vertical transmission during birth^37^^,^^38^ or horizontal transmission in childhood, while in low endemic areas like Northwest Europe, HBV infection is more often acquired at a later age.^37^ Considering the large differences between infant and adult immune responses,^39^ differences in ethnic origin could in part influence the outcomes reported in this SLR and limit the extrapolation of the results to other populations. Additionally, geographical differences in HBV genotype have been reported, which can potentially impact the severity of the disease, the progression of HBV infection, and treatment outcome.^37^ Consequently, information on the ethnicity and geography may provide valuable insights into other variables, such as duration of infection, mode of transmission and genotype. In this SLR, approximately one-third of the publications (34%) were conference abstracts with limited quantitative and baseline demographic data reported.

In the RETRACT-B study, a global study that followed patients with virally suppressed chronic HBV infection who were HBeAg negative, cumulative probability of HBsAg loss was 3% at 12 months and 13% at 48 months^33^; rates were higher among White vs Asian people, and in those with low HBsAg levels at EOT. The correlation between non-Asian race and low HBsAg levels with favorable outcomes was also shown in the CREATE study.^40^ HBsAg loss was observed in 4% of patients who stopped NA therapy (maximum follow-up of 48 weeks)^40^; lower levels of HBcrAg and HBsAg were associated with improved outcomes, and the authors proposed that these factors could be used for risk stratification. A recent SLR, which reviewed the data on novel viral and immune markers in treatment discontinuation studies proposed a different algorithm to determine whether NA should be discontinued, based on quantitative HBsAg (qHBsAg), HBcrAg, and HBV RNA stratified by HBeAg status.^41^ The study concluded that NA should be continued if EOT qHBsAg is ≥ 2 log, and NA cessation can be considered when the EOT qHBsAg is < 2 log in combination with HBV RNA <3 log or HBcrAg <4 log for initially HBeAg-positive patients.^41^ Validation of the different algorithms is warranted to identify the appropriate variables associated with successful treatment withdrawal.

It is generally accepted that the lower the HBsAg level, the greater the chance of success, but there is no defined threshold.^12^ Furthermore, some centers do not quantify HBsAg as it does not inform the treatment decision process. A recent Viewpoint article from the HBV Forum noted that EOT HBsAg levels were the most promising biomarker, but there was no agreement as to what threshold should be used to direct safe NA cessation.^42^ While no consensus could be reached on criteria for NA cessation in patients with detectable HBsAg, the authors agreed that NA cessation criteria should include HBsAg and HBV DNA loss together with normal or slightly elevated ALT levels.^42^ The authors also noted that lower HBcrAg levels were associated with higher HBsAg loss and reduced virological relapse after NA cessation.^42^ The RCT, STOP-NUC, which was published after the SLR searches were run, measured HBsAg loss rate following NA cessation in 166 HBeAg-negative patients.^43^ Cessation of treatment was associated with significantly higher rates of HBsAg loss (∼10%) compared with the group who continued treatment; this was mostly in patients with HBsAg levels below 1000 IU/mL and the outcome did not differ between TDF or ETV treatment. However, a limitation of this study is that it was focused on Caucasian participants. An additional study demonstrated that withdrawal of TDF after 4 years of treatment was associated with a low rate of HBsAg decline or loss, and ALT elevations were frequent; thus, cessation was not supported as a therapeutic strategy.^35^ In our study, 38% (56 of 145) studies did not even report the NA stopping criteria. Further work is needed to clearly define the subgroup of patients in whom this strategy could be beneficial, and how to identify and monitor them.

The lack of granularity in many publications regarding the definitions of an ALT flare, restart criteria, heterogeneity of data available and lack of baseline demographics make it difficult to provide strong recommendations on NA cessation. Due to this lack of granularity, data on the impact of HBV reactivation and ALT flares following NA cessation are sparse. Few studies in this SLR reported rates of severe outcomes such as HD, HF, and HRD. Rates of HD were generally low and the number of patients included was generally high, with 8631 in one study.^19^ Furthermore, rates of liver transplantation were not analyzed.

There were a limited number of prospective clinical studies identified and in those that were, they lacked data on key outcomes of interest such as virological or clinical relapse, retreatment, HBeAg-related and other clinical outcomes, and had limited data for HBsAg loss and biochemical relapse. Finally, the duration of NA treatment varied widely between studies with median durations ranging from 24 to 571.6 weeks.

These results also highlight areas where future research should be focused, and where overall clinical practice may be optimized. For example, the COIN-B trial (NCT04779970), is assessing the controlled interruption of NA treatment in adults with chronic HBV infection investigating both viral control and HBsAg loss; the trial is estimated to complete in late 2025.^44^ Clearer guidance on how to identify patients or specific populations where NA cessation are warranted, along with standardized definitions of relapse, retreatment criteria, and tools and scoring systems to facilitate the calculation of a risk benefit ratio. Besides clearer guidance on HBsAg thresholds, further data are required to evaluate the predictive value of other variables, such as HBcrAg and race, and variables related to race including duration of infection, mode of transmission and genotype, as there was only limited evidence available. In addition, studies reporting on the “resetting” of HBsAg levels, such as that included in the recently redefined partial cure definition,^45^ was beyond the scope of this SLR; however, these data would be useful when determining the full chronic HBV treatment landscape. Finally, while data identified in this SLR showed that cessation of TDF therapy was associated with a higher risk of clinical relapse than cessation of ETV, data were limited, warranting further study.

It is likely that future regimens to establish functional cure will consist of combinations of novel agents with or without NAs.^42^ As functional cure is defined as sustained HBsAg and HBV DNA loss after cessation of all treatments,^45^ NA cessation will therefore be an important part of assessing functional cure for novel regimens. Beyond NA cessation after HBsAg loss, different algorithms have been proposed, but lack validation.^33^^,^^40^, ^41^, ^42^ Data from emerging studies will be essential to validate these algorithms and allow for the establishment of a standard diagnostic algorithm for successful NA cessation in appropriate patients with chronic HBV infection in future. Owing to the risks associated with virological relapse, it would be important to develop standardized NA restart criteria that will allow trials to look at the benefits of NA cessation while safeguarding patients.

In addition, while NA cessation is included in guidance documents,^4^, ^5^, ^6^ this may not be done routinely or according to the guidelines in clinical practice owing to additional factors, such as patient or physician preference. The incidence of such self-cessation and its impact on patient outcomes is largely unknown and data are limited, though spontaneous NA cessation has been documented at high frequency, even with a lack of clear and consistent guidance. The decision to stop NA may also be influenced by access, ie, whether treatment is provided through a national health-care system or through private insurance. For example, in the present systemic literature review search, many studies were included from Taiwan, where there are reimbursement criteria that mandate stopping NA treatment at 3 years regardless of HBsAg levels, NA response or disease activity.

To achieve the World Health Organization target of elimination of viral hepatitis as a public health threat by 2030, some have recommended a “treat all” rather than “treat select” approach.^46^ As evidence regarding NA cessation is currently only available for patient populations in which treatment is recommended, evidence for NA cessation in populations not currently eligible for treatment would need to be generated to inform this “treat all” approach.

As patients can relapse many years after NA cessation, careful and long-term monitoring is required. However, the optimal follow-up time is not known and recommendations vary across patient types and across regions.^13^ Monitoring ensures that treatment can be restarted if needed, therefore minimizing risk of relapse or disease progression. Furthermore, standardization of retreatment criteria will also contribute towards mitigating the associated risks. In this SLR, rates of retreatment were only reported in 14 studies. Further research into when NA therapy should be restarted including predictive factors is warranted. This is supported by the recent need to re-evaluate the restart criteria in the REEF-2 trial during which despite the highly regulated conditions, a patient without preidentified risk factors developed HD and needed a liver transplant.^47^

## Conclusion

Taken together, this SLR confirms that NA cessation leads to high rates of relapse, and to HBsAg loss only in a small subset of patients. Low baseline HBsAg was a predictor of HBsAg loss, and lower rates of clinical relapse and virological relapse, but robust data were lacking regarding other potential predictors, such as HBV RNA, HBcrAg, and race. NA cessation criteria and stopping rules varied, though generally reflected the local guidelines; outcome definitions also varied, reflecting a lack of standardization in, for example, duration of treatment and monitoring, among other factors. Data are incomplete to provide recommendations for NA cessation and to appropriately stratify patients who may benefit from this approach in clinical practice as well as clinical trials. Clearer guidelines and tools to assess and monitor patients with chronic HBV infection are required to guide treatment decisions and drug development, and to optimize the risk: benefit profile of NA cessation.

## Supplementary materials

Supplementary M ethods
