# Supplementary material for: Nucleos(t)ide Analog Treatment Discontinuation in Chronic Hepatitis B Virus Infection: A Systematic Literature Review
Source: Gastro Hep Adv. 2024 Aug 23;4(1):100536. doi: 10.1016/j.gastha.2024.08.015 (PMC11714690; doi:10.1016/j.gastha.2024.08.015)
Supplement: Supplementary M — ethods [file mmc1.docx]

# Supplementary Materials

## Supplementary Methods

## Supplementary Table 1. Search terms for systematic literature review in Embase database.

1. **Initial search (inception to February 2020)**

| Facet | No. | Query | Hits |
| --- | --- | --- | --- |
| Disease | #1 | ‘chronic hepatitis b’/syn OR ‘chronic hepatitis b’/exp OR Chabut | 33,107 |
|  | #2 | chronic NEAR/4 (‘hepatitis b’ OR ‘hepatitis-b’ OR HBv) | 38,745 |
|  | #3 | #1 OR #2 | 40,855 |
| NAs | #4 | ‘lamivudine’/syn OR ‘adefovir dipivoxil’/syn OR ‘entecavir’/syn OR ‘telbivudine’/syn OR ‘tenofovir’/syn OR ‘clevudine’/syn OR ‘ana 380/lb80380’ OR ‘thymosin alpha1’/syn OR (nucleotide NEAR/1 analogues) OR (nucleoside NEAR/1 analogues) | 61,469 |
| Treatment cessation | #5 | ‘cessation’/syn OR (treatment NEAR/3 cessation) OR discontinuation OR (treatment NEAR/2 discontinuation) OR ‘treatment cessation’ OR (treatment NEAR/2 stop) | 209,422 |
| NAs + treatment cessation | #6 | #4 AND #5 | 2624 |
| **Combined** | **#7** | **#3 AND #6** | **728** |

1. **Updated search (January 2020 to July 2022)**

| Facet | No. | Query | Hits |
| --- | --- | --- | --- |
| Disease | #1 | ‘chronic hepatitis b’/syn OR ‘chronic hepatitis b’/exp OR CHB:ab,ti | 39,279 |
|  | #2 | chronic NEAR/4 (‘hepatitis b’ OR ‘hepatitis-b’ OR HBv) | 44,283 |
|  | #3 | #1 OR #2 | 46,927 |
| NAs | #4 | ‘lamivudine’/syn OR ‘adefovir dipivoxil’/syn OR ‘entecavir’/syn OR ‘telbivudine’/syn OR ‘tenofovir’/syn OR ‘clevudine’/syn OR ‘ana 380/lb80380’ OR ‘thymosin alpha1’/syn OR (nucleotide NEAR/1 analogues) OR (nucleoside NEAR/1 analogues) | 70,380 |
| Treatment cessation | #5 | ‘cessation’/syn OR (treatment NEAR/3 cessation) OR discontinuation OR (treatment NEAR/2 discontinuation) OR ‘treatment cessation’ OR (treatment NEAR/2 stop) | 247,568 |
| NAs + treatment cessation | #6 | #4 AND #5 | 3120 |
| Combined | #7 | #3 AND #6 | 851 |
| **From 2020 onwards** | **#8** | **#3 AND #6 AND [2020-2022]/py** | **120** |

EMBASE, Excerpta Medica Database.

## Supplementary Figure 1. Distribution of included studies by (A) study design, (B) sample size, (C) study setting and (D) country


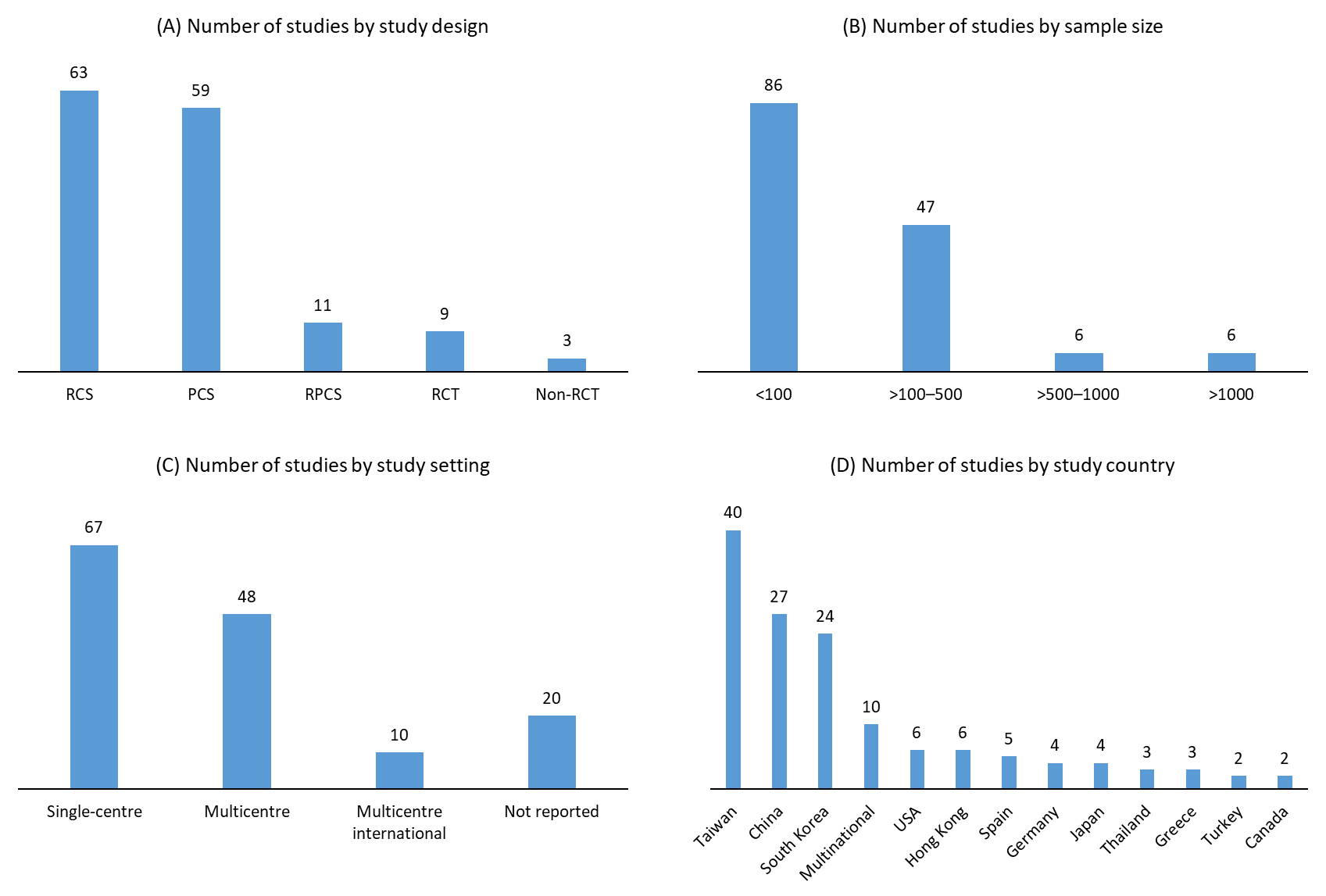


PCS, prospective cohort study; RCS, retrospective cohort study; RPCS, retrospective-prospective cohort study;
RCT, randomised controlled trial.

## Supplementary Table 2. Inclusion/exclusion criteria

| Domain | Inclusion criteria | Exclusion criteria |
| --- | --- | --- |
| **Population** | - Patients with chronic HBV infection who discontinued therapy with NAs | - Acute HBV - Any disease other than chronic HBV infection |
| **Intervention/**  **Comparators** | - Any NAs | - Other therapies for chronic HBV infection including interferons |
| **Outcomes** | - Categorical outcomes of interest:   - Virological relapse   - Clinical relapse   - Biochemical relapse   - Retreatment after discontinuation   - HBsAg-related outcomes incl. HBsAg loss   - HBeAg-related outcomes incl. HBeAg seroconversion, and   - Other clinical outcomes reported after  NA cessation | - Outcomes not of interest   - Other categorical outcomes other than those listed   - Continuous data for biomarkers at different timepoints after cessation of NAs |
| **Study design** | - Prospective cohort studies - Retrospective cohort studies - Cross-sectional studies - Clinical trials (RCTs, non-RCTs & single-arm trials) | - Case reports - Case series (up to 10 patients) |
| **Publication/ study type** | - Primary studies available as full journal publications - Conference abstracts 2020–2022 for AASLD, EASL and APASL - SLRs and TLRs providing information for objectives of interest were included for bibliography | Publication types:   - Narrative review articles - Editorials - Comments - Correspondence   Study types:   - Animal studies - In-vitro studies - Model-based studies |
| **Publication timeframe** | - Initial searches: Inception to January 2020 - Updated searches: January 2020 to July 2022 | - Not applicable |
| **Language** | - Only English language articles | - Non-English articles |
| **Geography** | - Any | - Not applicable |

AASLD, American Association for the Study of Liver Diseases; APASL, Asian-Pacific Association for the Study of the Liver; EASL, European Association for the Study of the Liver; HBV, hepatitis B virus; NA, nucleos(t)ide analogues; RCT, randomised controlled trial; SLR, systematic literature review; TLR, targeted literature review.

## Supplementary Table 3. Overview of included studies

| **Study name** | **Study design** | **Study country** | **Data collection period** | **Follow-up duration (wk)** | **Study population (At NA cessation)** | **Sample size (overall)** | **Sample size at (NA discontinued population)** |
| --- | --- | --- | --- | --- | --- | --- | --- |
| **Clinical trials** | | | | | | | |
| Berg 2017^1^ (FINITE, NCT01320943) | RCT | Germany | Apr 2011–Aug 2016 (Data collection period) | 144 wk (min FU) | HBeAg- | 42 | 21 |
| Brouwer 2015^2^ (ARES, NCT00877760) | RCT | Asia, Europe (14 centres across five countries) | 2009–2013 (Data collection period) | NR | HBeAg- | 175 | 22 |
| Dienstag 1999a^3^ | RCT | USA | May 1995–Aug 1997 (Data collection period) | 16 wk (min FU) | Mixed | 137 | 66 |
| Dienstag 1999b^4^ | Non-RCT | USA | NR | 17.3 (min FU)–52 wk (max FU) | HBeAg- | 24 | 7 |
| Feld 2021^5^ (HBRNIA, NCT01369212) | RCT | Canada, US | Nov 2012–Mar 2021 (Study duration) | 52 wk (min FU) | HBeAg- | 92 | 92 |
| Goulis 2008^6^ | RCT | Multicountry (names of countries NR) | NR | 24 wk (min FU) | Mixed | 286 | 25 |
| Johannessen 2021^7^ | RCT | Denmark, Ethiopia, Norway, Sweden | NR | 52 wk (min FU) | HBeAg- | 127 | 127 |
| Lai 2020^8^ | RCT | Hong Kong | Sep 2016–Mar 2018 (Data collection period) | 150 wk (min FU) | Mixed | 19 | 13 |
| Liem 2019^9^ (Toronto STOP, NCT01911156) | RCT | Canada | May 2016–May 2018 (Data collection period) | 72 wk (min FU) | HBeAg- | 67 | 45 |
| Sung 2005^10^ | RCT | China | NR | 52 wk (min FU) | Mixed | 47 | 47 |
| Wong 2018^11^ | Non-RCT | Hong Kong | NR | 206 wk (mean, SD: 14 wk) | HBeAg+ (immune tolerant) | 20 | 20 |
| Zhang 2013^12^ | Non-RCT | China | Jan 2006–Jun 2010 (Recruitment period) | ≤24 wk (min FU, group 1) and >24 wk (max FU, group 2) | HBeAg+ | 50 | 50 |
| **Observational studies** | | | | | | | |
| Abdurakhmanov 2020^13^ | PCS | Moscow | NR | 156 wk (min FU) | Mixed | 36 | 36 |
| Akuta 2005^14^ | RCS | Japan | Sep 1995–Dec 2002 (recruitment period) | 72.8 wk (median, range: 7.8–348.4 wk) | Mixed | 269 | 25 |
| Alizadeh 2006^15^ | RCS | Iran | Mar 2002–Mar 2004 (recruitment period) | 26 wk (min FU) | HBeAg- | 71 | 71 |
| Assawasuwannakit 2021^16^ | PCS | Thailand | NR–Jun 2021 (Data collection period) | 142 wk (median, IQR: 126–158 wk) | HBeAg- | 92 | 92 |
| Azhari 2020a^17^ | RCS | Canada | 2009–2020 (Data collection period) | NR | HBeAg- | 1337 | 47 |
| Brakenhoff 2020^18^ | PCS | USA | NR | NR | Mixed | 344 | 344 |
| Broquetas 2021^19^ | RPCS | Spain | Dec 2017–Oct 2019 (Data collection period) | 96 wk (min FU) | HBeAg- | 52 | 52 |
| Buti 2015^20^ | PCS | Spain | NR | 72 wk (min FU) | HBeAg- | 17 | 8 |
| Byun 2005^21^ | PCS | South Korea | NR | 26 wk (median, range: 4.3–125.6 wk) | HBeAg- | 132 | 132 |
| Chan 2011^22^ | RCS | China | 1999–2004 (Recruitment period) | 203.5 wk (mean, SD: 151.6, range: 4.3–502.3 wk) | HBeAg- | 53 | 53 |
| Chang 2015^23^ | RCS | Taiwan | NR | 389.7 (median, range: 13–731.8 wk); 385.8 (mean, SD: 167.6 wk) | Mixed | 318 | 263 |
| Chaung 2012^24^ | RCS | USA | Mar 1998–Nov 2010 (recruitment period) | 116.9 wk (median, range: 8.7–264.1 wk) | HBeAg- | 88 | 39 |
| Chen 2013a^25^ | PCS | Taiwan | 2008–2011 (Recruitment period) | NR | HBeAg- | 126 | 126 |
| Chen 2013b^26^ | PCS | China | NR | 52 wk (median, range: 8.7–311.8 wk) | HBeAg- | 54 | 54 |
| Chen 2013c^27^ | PCS | Taiwan | 2002–2007 (Recruitment period) | NR | HBeAg- | 190 | 190 |
| Chen 2014^28^ | RCS | China | 2008–2013 (Recruitment period) | 48 wk (median, range: 12–204 wk, mean 77.3) | HBeAg- | 39 | 39 |
| Chen 2015a^29^ | RCS | Taiwan | 2007–2011 (Rx period) | 90 wk (median, range: 52–284 wk) | HBeAg- | 1565 | 252 |
| Chen 2015b^30^ | RCS | Taiwan | 2004–2012 (Recruitment period) | NR | HBeAg- | 485 | 485 |
| Chen 2017^31^ | RCS | Taiwan | 2007–2013 (Recruitment period) | NR | HBeAg- | 414 | 414 |
| Chen 2018^32^ | RCS | Taiwan | 2011–2013 (Recruitment period) | 55 wk (median, range: 24–149, IQR: 36-85 wk) | HBeAg- | 143 | 143 |
| Chen 2021^33^ | PCS | Taiwan | NR | 16 wk (min FU) | HBeAg- | 124 | 93 |
| Chen 2022^34^ | PCS | China | NR | 52 wk (min FU) | HBeAg- | 64 | 64 |
| Chi 2015a^35^ | RCS | Canada, The Netherlands | 1998–Jan 2014 (Data collection period) | 84 wk (median, IQR: 27.3–182.7 wk) | HBeAg- | 94 | 94 |
| Chi2017^36^ | RCS | Canada, China, The Netherlands | 1997–2015 (Rx period) | 83.2 wk (median, IQR: 26.0–140.4 wk) | HBeAg- | 70 | 54 |
| Chi 2019^37^ | PCS | China | Nov 2012–Feb 2017 (Data collection period) | 130 wk (median, IQR: 52–182 wk) | HBeAg- | 100 | 100 |
| Dienstag 2003^38^ | PCS | Europe, North America, South Africa, The Middle East | Jan 1997–Oct 2000 (Data collection period) | 176.2 wk (median, range: 97.4–269.8 wk) | HBeAg- | 40 | 40 |
| Fan 2020a^39^ (EFFORT 1 [NCT00962533] and EFFORT 2 [NCT01529255], EFFORT 3 [NCT02826070]) | PCS | China | NR | 208 wk (min FU) | HBeAg- | 186 | 186 |
| Fang 2022^40^ | RCS | Taiwan | 2008-2016 (Recruitment period) | 228.1 wk (mean, SD: 99.1 wk) | HBeAg- | 243 | 243 |
| Fong 2015^41^ | RCS | USA | NR | 134.7 wk (median, range: 8.2–309.2; mean: 131.2 wk) | HBeAg- | 54 | 54 |
| Fung 2009^42^ | RCS | China | Jul 1994–Jul 2004 (recruitment period) | 86.6 wk (median, range: 8.7–307.4 wk) | HBeAg- | 101 | 22 |
| Gao 2020^43^ | RCS | China | Jul 2014–Dec 2015 (recruitment period) | 52 wk (min FU) | HBeAg- | 158 | 36 |
| Garcia-Lopez 2021^44^ | PCS | Spain | NR | 147.74 wk (median, IQR: 112.98–160.77 wk) | HBeAg- | 27 | 27 |
| Ge 2015^45^ | RCS | China | May 2005-Jul 2010 (Data collection period) | 38.9 wk (mean, SD: 40.6, range: 4.3–86.6 wk): virological relapse group  111.3 wk (mean, SD: 9.4, range: 104–156 wk): No virological relapse group | HBeAg- | 424 | 204 |
| Guerra 2022^46^ | PCS | Italy | NR | 69.5 wk (median, IQR 43.4–82.6 wk) | NR | 38 | 38 |
| He 2013^47^ | RCS | China | 2002–2008 (Recruitment period) | 24 wk (min FU) | HBeAg- | 66 | 66 |
| He 2014^48^ | RCS | China | 2002–2008 (Recruitment period) | 48 wk (min FU) | Mixed | 97 | 97 |
| Hirode 2022a^49^ (RETRACT-B) | PCS | Asia, Europe, and North America (Belgium, Canada, Germany, Greece, Hong Kong, Netherlands, Spain, Taiwan) | 2001–2020 (Recruitment period) | 80 wk (median, range: 34.2–171.2 wk) | HBeAg- | 1552 | 1552 |
| Hoener 2015^50^ | PCS | Germany | NR | 52 wk (min FU) | HBeAg- | 15 | 15 |
| Honkoop 2000^51^ | RPCS | The Netherlands | NR | 26 wk (min FU) | Mixed | 41 | 41 |
| Hsu 2018^52^ | PCS | Taiwan | NR | 109.1 wk (median, range: NR) | HBeAg- | 135 | 135 |
| Hsu 2021^53^ | RCS | Taiwan | Jan 2015–Dec 2018 (recruitment period) | 112.10 wk (median, range: NR) | NR | 10192 | 10192 |
| Huang 2014^54^ | PCS | Taiwan | Feb 2012–NR (Recruitment period) | 36.6 wk (median, range: 3–102 wk) | HBeAg- | 90 | 70 |
| Hung 2017^55^ | RCS | Taiwan | Aug 2002–Jan 2011 (Rx period) | 289.2 wk (mean, SD: 156.7 wk) | HBeAg- | 231 | 73 |
| Ito 2004^56^ | RPCS | Japan | 1996–2002 (Rx period) | 56.3 wk (median, range: 39–112.6 wk) | HBeAg- | 33 | 33 |
| Jackson 2022^57^ (HBV-STOP) | PCS | Australia | NR | 96 wk (min FU) | HBeAg- | 65 | 65 |
| Jang 2005^58^ | RCS | South Korea | Jan 2000–Jun 2003 (Rx period) | 124.7 wk (mean, SD: 68, range: 52–207.8 wk) | HBeAg+ | 73 | 35 |
| Jeng 2013^59^ | RPCS | Taiwan | NR | 48 wk (min FU) | HBeAg- | 95 | 95 |
| Jeng 2016a^60^ | RPCS | Taiwan | NR | 39 wk (median, range: 4–133 wk) | HBeAg- | 85 | 85 |
| Jeng 2018^61^ | PCS | Taiwan | NR | 155 wk (median, range: 2-614 wk) | HBeAg- | 1075 | 691 |
| Jeng 2021a^62^ | PCS | Taiwan | NR | 239.8 wk (median, range: 26–969.8 wk) | HBeAg- | 122 | 122 |
| Jeng 2021b^63^ | RCS | Taiwan | NR | 276.4 wk (median, range: NR) | HBeAg- | 275 | 275 |
| Jeng 2022^64^ | PCS | Taiwan | NR–Dec 2021 (Data collection period) | 344.14 wk (median, IQR: 260.71–427.57 wk) | HBeAg- | 691 | 691 |
| Jiang 2015^65^ | PCS | China | Sep 2005–Apr 2012 (recruitment period) | 26 wk (min FU) | HBeAg- | 72 | 72 |
| Jin 2012^66^ | RCS | South Korea | Jan 2007–Dec 2008 (recruitment period) | 125.6 wk (median, range: 8.7–186.2 wk) | Mixed | 138 | 138 |
| Jun 2016^67^ | RCS | South Korea | Mar 2008–Dec 2013 (discontinuation period) | 89.6 wk (mean, SD: 19.5 wk): non-relapse group 87.5 wk (mean, SD: 22.1 wk): Relapse group | HBeAg- | 58 | 58 |
| Jung 2009^68^ | RCS | South Korea | NR | 47.6 wk (median, range: 13–112.5 wk) | HBeAg- | 120 | 19 |
| Jung 2011^69^ | RCS | South Korea | NR | 52 wk (median, range: 13–129.9 wk) | HBeAg- | 36 | 19 |
| Jung 2016^70^ (QUIT, NCT01533051) | PCS | South Korea | Jun 2011–Sep 2013 (recruitment period) | 98.3 wk (median, IQR: 86.6–116 wk) | HBeAg- | 113 | 113 |
| Kaewdech 2020^71^ | PCS | Thailand | Feb 2018–Aug 2019 (recruitment period) | 48 wk (min FU) | HBeAg- | 97 | 92 |
| Kang 2017^72^ | RCS | South Korea | 1997–2014 (Recruitment period) | 298.8 wk (median, IQR: 114.7–459.0 wk) | HBeAg- | 240 | 157 |
| Karakaya 2017^73^ | RPCS | Turkey | 2007–2008 (Recruitment period) | 268 wk (median, range: 52–416 wk) | HBeAg- | 183 | 23 |
| Kim 2009^74^ | RCS | South Korea | NR | 168 wk (mean, range: 60.6-411.4) | HBeAg- | 376 | 55 |
| Kim 2013a^75^ | PCS | South Korea | 2003–2011 (Recruitment period) | 87.1 wk (mean, SD: 47.1 wk) | HBeAg- | 45 | 45 |
| Kim 2020^76^ | RCS | South Korea | Jan 2008–Jul 2018 (Data collection period) | 116.89 wk (median, IQR: 53.01–213.79 wk) | NR | 276 | 131 |
| Kranidioti 2019^77^ | PCS | Greece | Jul 2013–NR (data collection period) | 239.2 wk (median, range: 166.4–270.4 wk) | HBeAg- | 70 | 23 |
| Kuo 2019^78^ | RPCS | Taiwan | 2007–2014 (Recruitment period) | 131 wk (median, range: 52 wk): ETV group 65 wk (median, range: 34–202 wk): TDF group | HBeAg- | 773 | 507 |
| Kuo 2021^79^ | RCS | Taiwan | 2011–2016 (Recruitment period) | 141.9 wk (mean, SD: 73.4, range: 28–338 wk) | HBeAg- | 521 | 185 |
| Lai 2021^80^ | RCS | Taiwan | Oct 2003–Jan 2019 (recruitment period) | 108 wk (median, range: 44.7–206.6 wk): non-SAE group 85 wk (median, range: 40–161.5 wk): SAE group | HBeAg- | 234 | 234 |
| Lee 2002^81^ | RCS | South Korea | Jan 1997–Sep 2000 (Rx period) | NR | HBeAg- | 124 | 42 |
| Lee 2010^82^ | RCS | South Korea | Jan 1999–Aug 2004 (recruitment period) | 229.5 wk (mean, range: 103.9–389.7 wk) | HBeAg- | 178 | 178 |
| Lee 2015^83^ | RCS | Taiwan | 2012–2014 (Data collection period) | 52 wk (mean, SD: 31 wk) | Mixed | 105 | 93 |
| Lee 2016^84^ | RCS | South Korea | NR | 90.1 wk (mean, SD: 86.2) | HBeAg- | 44 | 44 |
| Lens 2022^85^ | PCS | Spain | NR | 147.73 wk (median, IQR: 112.98–160.77 wk) | HBeAg- | 21 | 21 |
| Li 2021^86^ | RPCS | China | Dec 2001–Jan 2020 (recruitment period) | 260 wk (min FU): N=81 156 wk (min FU): N=101 | HBeAg- | 190 | 190 |
| Liang 2011^87^ | RCS | China | May 2004–Oct 2010 (Data collection period) | 36.4 wk (mean, SD: 26.0 wk) | HBeAg- | 84 | 84 |
| Lin 2016^88^ | RCS | Taiwan | Jan 2009–Dec 2010 (recruitment period) | 52 wk (min FU) | Mixed | 266 | 88 |
| Liu 2018^89^ | PCS | China | 2001–2015 (Recruitment period) | 259.8 wk (median, range: 52–311.8 wk) | HBeAg- | 223 | 223 |
| Liu 2020^90^ | RPCS | Taiwan | NR | 55.6 wk (median, range: NR) | HBeAg- | 1242 | 1242 |
| Liu 2021a^91^ | PCS | Taiwan | NR | NR | HBeAg- | 1238 | 1238 |
| Liu 2021b^92^ | RCS | Taiwan | NR | 104 wk (min FU) | HBeAg- | 214 | 214 |
| Ma 2019^93^ | RCS | Taiwan | 2008–2015 (Recruitment period) | 52 wk (min FU) | HBeAg- | 535 | 535 |
| Manolakopoulos 2021^94^ | PCS | Greece | Apr 2012–Nov 2015 (recruitment period) | 282.44 wk (median, range: 156.42–378.03 wk) | HBeAg- | 57 | 57 |
| Nagata 2016^95^ | RCS | Japan | Dec 1999–Jun 2020 (recruitment period) | NR | HBeAg- | 94 | 33 |
| Ohlendorf 2022^96^ (ABX-203, NCT02249988) | PCS | Germany | NR | 24 wk | HBeAg- | 154 | 154 |
| Paik 2010^97^ | PCS | South Korea | Dec 1997–Jan 2004 (Rx period) | 176.7 wk (mean, SD: 98.3; range: 52–415.7 wk) | HBeAg- | 50 | 43 |
| Pan 2015^98^ | RPCS | China | Jan 2000–Jun 2014 (Rx period) | 498 wk (median, range: 208–528 wk) | HBeAg- | 368 | 86 |
| Papatheodoridis 2017a^99^ | RCS | NR | NR | 65.0 wk (median, IQR: 56.3 wk) | HBeAg- | 130 | 130 |
| Papatheodoridis 2018a^100^ (DARING-B) | PCS | Greece | 2015–2016 (Recruitment period) | 77.94 wk (median, range: NR) | HBeAg- | 60 | 57 |
| Park 2016^101^ | RCS | South Korea | Jan 2006-Dec 2012 (Rx period) | NR | Mixed | 804 | 226 |
| Patwardhan 2014^102^ | RCS | USA | 1999–2009 (Recruitment period) | 156 wk (median, range: 104–156 wk) | HBeAg- | 435 | 33 |
| Peng 2015^103^ | PCS | China | Jan 2003-May 2014 (Data collection period) | NR | HBeAg- | 65 | 65 |
| Peng 2021a^104^ | PCS | Taiwan | NR | 104 wk (min FU) | HBeAg+ | 125 | 125 |
| Peng 2021b^105^ | PCS | Taiwan | NR | 104 wk (min FU) | HBeAg+ | 125 | 125 |
| Petersen 2011a^106^ | RCS | Germany | NR | NR | HBeAg- | 32 | 32 |
| Petersen 2013a^107^ | RPCS | NR | NR | 199.2 wk (median, range: NR) | HBeAg- | 14 | 14 |
| Pocurull 2022^108^ | PCS | Spain | NR | 121.67 wk (median, range 104.2–204.2 wk) | HBeAg- | 96 | 96 |
| Qiu 2016^109^ | RCS | China | Jan 2009–Dec 2011 (recruitment period) | 52 wk (min FU) | HBeAg- | 112 | 112 |
| Ridruejo 2014^110^ | RCS | Argentina | Jan 2005–NR (Rx period) | 66 wk (median, IQR: 33–128 wk) | Mixed | 169 | 36 |
| Ryu 2003^111^ | PCS | South Korea | NR | 69.3 wk (median, range: 52–134.2 wk) | HBeAg- | 85 | 61 |
| Sah 2019a^112^ | PCS | Turkey | Jan 2015–Jan 2017 (recruitment period) | 125.6 wk (median, range: 52–173.2 wk) | HBeAg- | 36 | 36 |
| Santos 2019^113^ | RCS | Portugal | NR | 121.2 wk (median, IQR: 91–130 wk) | HBeAg- | 47 | 47 |
| Sattayalertyanyong 2020a^114^ | PCS | Thailand | NR | 24 wk (min FU, preliminary analysis/report) | HBeAg- | 86 | 60 |
| Seto 2015^115^ | PCS | Hong Kong | Feb 2012–Sep 2012 (recruitment period) | 48 wk (min FU) | HBeAg- | 184 | 184 |
| Seto 2016^116^ | RCS | Hong Kong | Jul 1999–May 2013 (recruitment period) | 174.1 wk (median, IQR: 92.7–254.2 wk) | HBeAg- | 102 | 22 |
| Seto 2021^117^ | PCS | Hong Kong | Jan 2016–Oct 2018 (recruitment period) | 48 wk (min FU) | HBeAg- | 299 | 114 |
| Shin 2005^118^ | RCS | South Korea | Mar 1998–May 2003 (Rx period) | 26.4 wk (mean, range: 4.3–95.3 wk) | HBeAg- | 192 | 121 |
| Sohn 2014^119^ | RCS | South Korea | Nov 2004–May 2010 (Rx period) | 95 wk (median, range: NR) | HBeAg- | 2736 | 95 |
| Song 2012^120^ | RCS | South Korea | Feb 2007–Jan 2010 (recruitment period) | 78.8 wk (median, range: 22.1–205.7 wk) | HBeAg- | 48 | 48 |
| Song 2018a^121^ | RCS | South Korea | NR | NR | Mixed | 359 | 359 |
| Song 2021^122^ | RCS | South Korea | Jun 1997–Feb 2015 (recruitment period) | 318.5 wk (median, range: NR) | Mixed | 488 | 488 |
| Sonneveld 2022a^123^ (CREATE) | PCS | Asia, Europe, Taiwan | NR | 102.5 wk (median, IQR 48–244 wk) | HBeAg- | 1216 | 1216 |
| Sonneveld 2022b^124^ | PCS | Greece, Germany, Hong Kong, Japan, Korea, Spain, UK, USA | NR | 74 wk (median, IQR 48-179 wk) | HBeAg- | 243 | 243 |
| Su 2018^125^ | PCS | Taiwan | 2012–2017 (Recruitment period) | 138.6 wk (median, patients with clinical relapse) and 151.6 wk (median, patients without clinical relapse) | HBeAg- | 172 | 100 |
| Su 2020^126^ | RCS | Taiwan | Oct 2003–Dec 2011 (Rx period) | 72 wk (min FU) | NR | 17262 | 8631 |
| Su 2021^127^ | PCS | Taiwan | Oct 2012–2021 (Data collection period) | 52 wk (min FU) | NR | 144 | 144 |
| Suh 2012^128^ | PCS | South Korea | Jun 1998–Dec 2010 (recruitment period) | 138.6 wk (mean, SD: 104 wk) | Mixed | 81 | 81 |
| Tseng 2018^129^ | PCS | Taiwan | Aug 2011–Jun 2016 (Data collection period) | 90.9 wk (mean, IQR: 52–129.9 wk) | HBeAg- | 82 | 82 |
| Tseng 2020^130^ | RCS | Taiwan | 2007–2016 (Rx period) | 87 wk (median, IQR: 48–161 wk): Development group (n=135) 126 wk (median, IQR: 61–214 wk): Validation group (n=108) | HBeAg- | 243 | 243 |
| Tseng 2022^131^ | RCS | Taiwan | 2008–2017 (Recruitment period) | 130 wk (min FU) | HBeAg- | 504 | 504 |
| Tsuge 2013^132^ | RCS | Japan | NR | 269 wk (mean, range: 73–508 wk) | Mixed | 36 | 36 |
| Tuefferd 2020^133^ | PCS | Taiwan | NR | 104 wk (min FU) | HBeAg- | 186 | 186 |
| Tzu-Ning 2021^134^ | RCS | Taiwan | NR | 104 wk (min FU) | HBeAg- | 472 | 472 |
| Wang 2010^135^ | PCS | China | NR | 103.9 wk (median, range: 8.7–363.7 wk): HBeAg seroconversion group 103.9 wk (median, range: 8.7–285.8 wk): HBeAg loss group | HBeAg- | 125 | 125 |
| Wang 2020^136^ | PCS | China | NR | 48 wk (min FU) | HBeAg+ | 626 | 626 |
| Wang 2022^137^ | RCS | China | Jun 2020–Jan 2021 (recruitment period) | 52 wk (min FU) | HBeAg- | 64 | 64 |
| Wong 2008^138^ | PCS | Hong Kong | Nov 1999–Sep 2004 (recruitment period) | 338 wk (median, range: 114.4–410.8 wk) | Mixed | 76 | 16 |
| Xie 2022^139^ | PCS | China | NR | 104 wk (min FU) | HBeAg- | 158 | 158 |
| Xu 2021^140^ (NCT02883647) | PCS | China | Jan 2014–Dec 2017 (Study period) Dec 2013–Dec 2018 (recruitment period) | 96 wk (min FU) | HBeAg- | 103 | 74 |
| Xu 2022^141^ | PCS | China | NR | 260 wk (min FU) | NR | 62 | 62 |
| Yao 2017^142^ | RCS | Taiwan | 2002–2011 (Rx and discontinuation period) | NR | HBeAg- | 365 | 119 |
| Yen 2018^143^ | PCS | Taiwan | Jan 2015–Jan 2018 (recruitment period) | 45.9 wk (median, IQR: 24.2–31.2 wk) | HBeAg- | 178 | 178 |
| Yoon 2005^144^ | RCS | South Korea | Jul 1998–Dec 2003 (recruitment period) | 176.2 wk (mean, range: 52–324.8 wk) | HBeAg- | 461 | 114 |
| Zhang 2017^145^ | PCS | China | 2010–NR (Recruitment period) | NR | HBeAg- | 138 | 138 |

FU, follow-up; HBeAg, hepatitis B e-antigen; HBeAg-, hepatitis B e-antigen negative; HBeAg+, hepatitis B e-antigen positive; IQR, interquartile range; max, maximum; min, minimum; Mixed, both HBeAg-negative
and HBeAg-positive; NR, not reported; PCS, prospective cohort study; RCS, retrospective cohort study; RPCS, retrospective-prospective cohort study; RCT, randomised controlled trial; Rx, treatment; Non-RCT,
non-randomised controlled trial; SD, standard deviation; SAE, severe acute exacerbation; wk, week.

## Supplementary Table 4. Risk of bias within individual randomised controlled clinical trials using a) the NICE checklist, b) the Risk Of Bias In Non-randomised Studies (ROBINS-I) checklist for non-RCTs, and c) the Newcastle-Ottawa Scale (NOS) checklist for observational studies

**a)**

| **Study name** | **Overall rating** | **Random sequence generation** | **Allocation concealment** | **Comparability of groups** | **Blinding** | **Imbalance in dropouts** | **Selective reporting** | **Incomplete reporting** |
| --- | --- | --- | --- | --- | --- | --- | --- | --- |
| **Study name** |  | **Low/Unclear/ High risk of bias?** | **Low/Unclear/ High risk of bias?** | **Low/Unclear/ High risk of bias?** | **Low/Unclear/ High risk of bias?** | **Low/Unclear/ High risk of bias?** | **Low/Unclear/ High risk of bias?** | **Low/Unclear/High risk of bias?** |
| Berg 2017^1^ | Unclear | Unclear risk | Unclear risk | Unclear risk | High risk | Unclear risk | Low risk | Low risk |
| Brouwer 2015^2^ | Low | Low risk | Low risk | Low risk | High risk | Low risk | Low risk | Low risk |
| Dienstag 1999a^3^ | Low | Unclear risk | Unclear risk | Low risk | Low risk | Low risk | Low risk | Low risk |
| Feld 2021^5^ | Unclear | Unclear risk | Unclear risk | Unclear risk | High risk | Unclear risk | Low risk | Unclear risk |
| Goulis 2008^6^ | Unclear | Unclear risk | Unclear risk | Unclear risk | Low risk | Unclear risk | Low risk | Unclear risk |
| Johannessen 2021^7^ | Unclear | Unclear risk | Unclear risk | Unclear risk | High risk | Unclear risk | Low risk | Unclear risk |
| Lai 2020^8^ | Unclear | Low risk | Unclear risk | Unclear risk | Unclear risk | Low risk | Low risk | Unclear risk |
| Liem 2019^9^ | Low | Low risk | Low risk | Low risk | High risk | Low risk | Low risk | High risk |
| Sung 2005^10^ | Unclear | Unclear risk | Unclear risk | Unclear risk | Unclear risk | Low risk | Low risk | Unclear risk |

NICE, National Institute for Health and Care Excellence.

**b)**

| **Study name** | **Dienstag 1999b** | **Wong 2018** | **Zhang 2013** |
| --- | --- | --- | --- |
| **Overall judgement (Low risk/Moderate risk/Serious risk/Critical risk/No information)** | Low risk | Low risk | Low risk |
| **Questions establishing need to assess confounding** |  |  |  |
| 1.1. Is there potential for confounding of the effect of intervention in this study? (Y/PY/PN/NO) | PN | PN | PN |
| 1.2. Was the analysis based on splitting participants’ follow-up time according to intervention received? (NA/Y/PY/PN/NO/NI) | Not App. | Not App. | Not App. |
| 1.3. If Y/PY to 1.2: Were intervention discontinuations or switches likely to be related to factors that are prognostic for the outcome? (NA/Y/PY/PN/NO/NI) | Not App. | Not App. | Not App. |
| **Questions relating to baseline confounding only** |  |  |  |
| 1.4. Did the authors use an appropriate analysis method that controlled for all the important confounding domains? (NA/Y/PY/PN/NO/NI) | Not App. | Not App. | Not App. |
| 1.5 If Y/PY to 1.4: Were confounding domains that were controlled for measured validly and reliably by the variables available in this study? (NA/Y/PY/PN/NO/NI) | Not App. | Not App. | Not App. |
| 1.6. Did the authors control for any post-intervention variables that could have been affected by the intervention? (NA/Y/PY/PN/NO/NI) | Not App. | Not App. | Not App. |
| **Questions relating to time-varying confounding** |  |  |  |
| 1.7. If Y/PY to 1.3: Did the authors use an appropriate analysis method that controlled for all the important confounding domains and for time-varying confounding? (NA/Y/PY/PN/NO/NI) | Not App. | Not App. | Not App. |
| 1.8. If Y/PY to 1.7: Were confounding domains that were controlled for measured validly and reliably by the variables available in this study? (NA/Y/PY/PN/NO/NI) | Not App. | Not App. | Not App. |
| **Bias due to confounding: Risk of bias judgement (Low/Moderate/Serious/Critical/NI)** | Low | Low | Low |
| **Bias in selection of participants into the study** |  |  |  |
| 2.1. Was selection of participants into the study (or into the analysis) based on participant characteristics observed after the start of intervention? If N/PN to 2.1: go to 2.4 (Y/PY/PN/NO/NI) | No | No | No |
| 2.2. If Y/PY to 2.1: Were the post-intervention variables that influenced selection likely to be associated with intervention? (NA/Y/PY/PN/NO/NI) | Not App. | Not App. | Not App. |
| 2.3 If Y/PY to 2.2: Were the post-intervention variables that influenced selection likely to be influenced by the outcome or a cause of the outcome? (NA/Y/PY/PN/NO/NI) | Not App. | Not App. | Not App. |
| 2.4. Do start of follow-up and start of intervention coincide for most participants? (Y/PY/PN/NO/NI) | Y | Y | Y |
| 2.5. If Y/PY to 2.2 and 2.3, or N/PN to 2.4: Were adjustment techniques used that are likely to correct for the presence of selection biases? (NA/Y/PY/PN/NO/NI) | Not App. | Not App. | Not App. |
| **Bias in selection of participants into the study: risk of bias judgement (Low/Moderate/Serious/Critical/NI)** | Low | Low | Low |
| **Bias in classification of interventions** |  |  |  |
| 3.1 Were intervention groups clearly defined? (Y/PY/PN/NO/NI) | Y | Y | Y |
| 3.2 Was the information used to define intervention groups recorded at the start of the intervention? (Y/PY/PN/NO/NI) | Y | Y | Y |
| 3.3 Could classification of intervention status have been affected by knowledge of the outcome or risk of the outcome? (Y/PY/PN/NO/NI) | No | No | No |
| **Bias in classification of interventions: Risk of bias judgement (Low/Moderate/Serious/Critical/NI)** | Low | Low | Low |
| **For studies where the aim is to assess the effect of ASSIGNMENT to intervention** |  |  |  |
| 4.1. Were there deviations from the intended intervention beyond what would be expected in usual practice? (Y/PY/PN/NO/NI) | No | No | No |
| 4.2. If Y/PY to 4.1: Were these deviations from intended intervention unbalanced between groups and likely to have affected the outcome? (NA/Y/PY/PN/NO/NI) | Not App. | Not App. | Not App. |
| For studies where the aim is to assess the effect of ADHERENCE to intervention |  |  |  |
| 4.3. Were important co-interventions balanced across intervention groups? (Y/PY/PN/NO/NI) | Not App. | Not App. | Not App. |
| 4.4. Was the intervention implemented successfully for most participants? (Y/PY/PN/NO/NI) | Not App. | Not App. | Not App. |
| 4.5. Did study participants adhere to the assigned intervention regimen? (Y/PY/PN/NO/NI) | Not App. | Not App. | Not App. |
| 4.6. If N/PN to 4.3, 4.4 or 4.5: Was an appropriate analysis used to estimate the effect of starting and adhering to the intervention? (NA/Y/PY/PN/NO/NI) | Not App. | Not App. | Not App. |
| **Bias due to deviations from intended interventions: Risk of bias judgement (Low/Moderate/Serious/Critical/NI)** | Low | Low | Low |
| **Bias due to missing data** |  |  |  |
| 5.1 Were outcome data available for all, or nearly all, participants? (Y/PY/PN/NO/NI) | Y | Y | Y |
| 5.2 Were participants excluded due to missing data on intervention status? (Y/PY/PN/NO/NI) | No | No | No |
| 5.3 Were participants excluded due to missing data on other variables needed for the analysis? (Y/PY/PN/NO/NI) | No | No | No |
| 5.4 If PN/N to 5.1, or Y/PY to 5.2 or 5.3: Are the proportion of participants and reasons for missing data similar across interventions? (NA/Y/PY/PN/NO/NI) | Not App. | Not App. | Not App. |
| 5.5 If PN/N to 5.1, or Y/PY to 5.2 or 5.3: Is there evidence that results were robust to the presence of missing data? (NA/Y/PY/PN/NO/NI) | Not App. | Not App. | Not App. |
| **Bias due to missing data: Risk of bias judgement (Low/Moderate/Serious/Critical/NI)** | Low | Low | Low |
| **Bias in measurement of outcomes** |  |  |  |
| 6.1 Could the outcome measure have been influenced by knowledge of the intervention received? (Y/PY/PN/NO/NI) | NI | NI | NI |
| 6.2 Were outcome assessors aware of the intervention received by study participants? (Y/PY/PN/NO/NI) | NI | NI | NI |
| 6.3 Were the methods of outcome assessment comparable across intervention groups? (Y/PY/PN/NO/NI) | No | No | No |
| 6.4 Were any systematic errors in measurement of the outcome related to intervention received? (Y/PY/PN/NO/NI) | NI | NI | NI |
| **Bias in measurement of outcomes: Risk of bias judgement (Low/Moderate/Serious/Critical/NI)** | Moderate | Moderate | Moderate |
| **Bias in selection of the reported result** |  |  |  |
| 7.1. Is the reported effect estimate likely to be selected, based on the results, from multiple outcome measurements within the outcome domain? (Y/PY/PN/NO/NI) | No | No | No |
| 7.2 Is the reported effect estimate likely to be selected, based on the results, from multiple analyses of the intervention-outcome relationship? (Y/PY/PN/NO/NI) | No | No | No |
| 7.3 Is the reported effect estimate likely to be selected, based on the results, from different subgroups? (Y/PY/PN/NO/NI) | No | No | No |
| **Bias in selection of the reported result: Risk of bias judgement** | Low | Low | Low |

Not App., not applicable; NI, no information; non-RCT, non-randomised controlled trial; PN, probably no; PY, probably yes; ROBINS-I, Risk Of Bias In Non-randomised Studies - of Interventions; Y, yes.

**c)**

| **Study name** | **Total out of nine stars** | **Representativeness of the cohort*** | **Selection of the non-exposed in the cohort**** | **Ascertainment of exposure***** | **Demonstration that outcome of interest was not present at start of study^#^** | **Comparability of subjects in cohort based on the design or analysis^$^** | **Assessment of outcome^** | **Was follow up long enough for outcomes to occur^$$^** | **Adequacy of follow-up of cohorts^^** |
| --- | --- | --- | --- | --- | --- | --- | --- | --- | --- |
| Abdurakhmanov 2020^13^ | **7** | 0 | 0 | 1 | 1 | 2 | 1 | 1 | 1 |
| Akuta 2005^14^ | **8** | 1 | 1 | 1 | 1 | 1 | 1 | 1 | 1 |
| Alizadeh 2006^15^ | **3** | 0 | 0 | 1 | 1 | 0 | 1 | 0 | 0 |
| Assawasuwannakit 2021^16^ | **6** | 1 | 1 | 1 | 1 | 1 | 0 | 1 | 0 |
| Azhari 2020^17^ | **5** | 0 | 0 | 1 | 1 | 1 | 1 | 0 | 1 |
| Brakenhoff 2020^18^ | **3** | 0 | 0 | 1 | 1 | 0 | 0 | 1 | 0 |
| Broquetas 2021^19^ | **7** | 0 | 1 | 1 | 1 | 1 | 1 | 1 | 1 |
| Buti 2015^20^ | **6** | 1 | 0 | 1 | 1 | 0 | 1 | 1 | 1 |
| Byun 2005^21^ | **5** | 1 | 1 | 1 | 1 | 0 | 0 | 1 | 0 |
| Chan 2011^22^ | **3** | 0 | 0 | 1 | 1 | 0 | 0 | 1 | 0 |
| Chang 2015^23^ | **6** | 1 | 1 | 1 | 1 | 0 | 0 | 1 | 1 |
| Chaung 2012^24^ | **4** | 1 | 1 | 1 | 1 | 0 | 0 | 0 | 0 |
| Chen 2013a^25^ | **3** | 1 | 0 | 0 | 1 | 0 | 0 | 1 | 0 |
| Chen 2013b^26^ | **3** | 0 | 0 | 1 | 1 | 0 | 0 | 1 | 0 |
| Chen 2013c^27^ | **4** | 0 | 0 | 1 | 1 | 1 | 1 | 0 | 0 |
| Chen 2014^28^ | **7** | 1 | 0 | 1 | 1 | 2 | 1 | 1 | 0 |
| Chen 2015a^29^ | **7** | 0 | 1 | 1 | 1 | 2 | 1 | 1 | 0 |
| Chen 2015b^30^ | **7** | 0 | 1 | 1 | 1 | 1 | 1 | 1 | 1 |
| Chen 2017^31^ | **7** | 0 | 0 | 1 | 1 | 2 | 1 | 1 | 1 |
| Chen 2018^32^ | **5** | 0 | 0 | 1 | 1 | 1 | 1 | 0 | 1 |
| Chen 2021^33^ | **5** | 0 | 0 | 1 | 1 | 1 | 1 | 0 | 1 |
| Chen 2022^34^ | **5** | 0 | 0 | 1 | 1 | 1 | 1 | 0 | 1 |
| Chi 2015a^35^ | **7** | 1 | 1 | 1 | 1 | 1 | 0 | 1 | 1 |
| Chi 2017^36^ | **3** | 0 | 0 | 1 | 1 | 0 | 0 | 1 | 0 |
| Chi 2019^37^ | **5** | 0 | 0 | 1 | 1 | 1 | 0 | 1 | 1 |
| Dienstag 2003^38^ | **7** | 1 | 0 | 1 | 1 | 1 | 1 | 1 | 1 |
| Fan 2020^39^ | **8** | 1 | 0 | 1 | 1 | 2 | 1 | 1 | 1 |
| Fang 2022^40^ | **8** | 1 | 1 | 1 | 1 | 1 | 1 | 1 | 1 |
| Fong 2015^41^ | **3** | 0 | 0 | 1 | 1 | 0 | 0 | 1 | 0 |
| Fung 2009^42^ | **6** | 0 | 0 | 1 | 1 | 1 | 1 | 1 | 1 |
| Gao 2020^43^ | **5** | 0 | 0 | 1 | 1 | 0 | 1 | 1 | 1 |
| Garcia-Lopez 2021^44^ | **3** | 0 | 0 | 1 | 1 | 0 | 0 | 1 | 0 |
| Ge 2015^45^ | **5** | 1 | 1 | 1 | 1 | 0 | 0 | 1 | 0 |
| Guerra 2022^46^ | **3** | 0 | 0 | 1 | 1 | 0 | 0 | 1 | 0 |
| He 2013^47^ | **6** | 1 | 0 | 1 | 1 | 0 | 1 | 1 | 1 |
| He 2014^48^ | **5** | 0 | 0 | 1 | 1 | 0 | 1 | 1 | 1 |
| Hirode 2022a^49^ | **8** | 1 | 0 | 1 | 1 | 2 | 1 | 1 | 1 |
| Hoener 2015^50^ | **6** | 1 | 1 | 1 | 1 | 0 | 0 | 1 | 1 |
| Honkoop 2000^51^ | **5** | 0 | 0 | 1 | 1 | 1 | 1 | 0 | 1 |
| Hsu 2018^52^ | **3** | 0 | 0 | 1 | 1 | 0 | 0 | 1 | 0 |
| Hsu 2021^53^ | **6** | 1 | 0 | 1 | 1 | 1 | 1 | 1 | 0 |
| Huang 2014^54^ | **5** | 1 | 0 | 1 | 1 | 1 | 1 | 0 | 0 |
| Hung 2017^55^ | **8** | 1 | 1 | 1 | 1 | 1 | 1 | 1 | 1 |
| Ito 2004^56^ | **8** | 0 | 1 | 1 | 1 | 2 | 1 | 1 | 1 |
| Jackson 2022^57^ | **3** | 0 | 0 | 1 | 1 | 0 | 0 | 1 | 0 |
| Jang 2005^58^ | **8** | 0 | 1 | 1 | 1 | 2 | 1 | 1 | 1 |
| Jeng 2013^59^ | **2** | 0 | 0 | 1 | 1 | 0 | 0 | 0 | 0 |
| Jeng 2016a^60^ | **6** | 0 | 1 | 1 | 1 | 1 | 1 | 0 | 1 |
| Jeng 2018^61^ | **5** | 1 | 1 | 1 | 1 | 0 | 0 | 1 | 0 |
| Jeng 2021a^62^ | **5** | 1 | 1 | 1 | 1 | 0 | 0 | 1 | 0 |
| Jeng 2021b^63^ | **7** | 1 | 0 | 1 | 1 | 2 | 1 | 1 | 0 |
| Jeng 2022^64^ | **6** | 1 | 1 | 1 | 1 | 0 | 0 | 1 | 1 |
| Jiang 2015^65^ | **6** | 1 | 1 | 1 | 1 | 0 | 0 | 1 | 1 |
| Jin 2012^66^ | **5** | 0 | 0 | 1 | 1 | 0 | 1 | 1 | 1 |
| Jun 2016^67^ | **3** | 0 | 0 | 1 | 1 | 0 | 0 | 1 | 0 |
| Jung 2009^68^ | **6** | 1 | 1 | 1 | 1 | 0 | 0 | 1 | 1 |
| Jung 2011^69^ | **4** | 0 | 0 | 1 | 1 | 0 | 0 | 1 | 1 |
| Jung 2016^70^ | **7** | 1 | 0 | 1 | 1 | 2 | 1 | 1 | 0 |
| Kaewdech 2020^71^ | **7** | 1 | 0 | 1 | 1 | 2 | 1 | 0 | 1 |
| Kang 2017^72^ | **8** | 1 | 0 | 1 | 1 | 2 | 1 | 1 | 1 |
| Karakaya 2017^73^ | **5** | 0 | 0 | 1 | 1 | 0 | 1 | 1 | 1 |
| Kim 2009^74^ | **7** | 0 | 0 | 1 | 1 | 2 | 1 | 1 | 1 |
| Kim 2013a^75^ | **7** | 1 | 0 | 1 | 1 | 1 | 1 | 1 | 1 |
| Kim 2020^76^ | **8** | 1 | 1 | 1 | 1 | 1 | 1 | 1 | 1 |
| Kranidioti 2019^77^ | **6** | 0 | 1 | 1 | 1 | 0 | 1 | 1 | 1 |
| Kuo 2019^78^ | **5** | 0 | 0 | 1 | 1 | 2 | 0 | 1 | 0 |
| Kuo 2021^79^ | **3** | 0 | 0 | 1 | 1 | 0 | 0 | 1 | 0 |
| Lai 2021^80^ | **8** | 0 | 1 | 1 | 1 | 2 | 1 | 1 | 1 |
| Lee 2002^81^ | **6** | 1 | 0 | 1 | 1 | 0 | 1 | 1 | 1 |
| Lee 2010^82^ | **6** | 1 | 0 | 1 | 1 | 1 | 1 | 1 | 0 |
| Lee 2015^83^ | **7** | 0 | 1 | 1 | 1 | 1 | 1 | 1 | 1 |
| Lee 2016^84^ | **6** | 1 | 0 | 1 | 1 | 0 | 1 | 1 | 1 |
| Lens 2022^85^ | **3** | 0 | 0 | 1 | 1 | 0 | 0 | 1 | 0 |
| Li 2021^86^ | **6** | 0 | 0 | 1 | 1 | 1 | 1 | 1 | 1 |
| Liang 2011^87^ | **4** | 1 | 1 | 1 | 1 | 0 | 0 | 0 | 0 |
| Lin 2016^88^ | **6** | 0 | 1 | 1 | 1 | 0 | 1 | 1 | 1 |
| Liu 2018^89^ | **5** | 1 | 1 | 1 | 1 | 0 | 0 | 1 | 0 |
| Liu 2020^90^ | **7** | 1 | 1 | 1 | 1 | 0 | 1 | 1 | 1 |
| Liu 2021a^91^ | **6** | 0 | 0 | 1 | 1 | 1 | 1 | 1 | 1 |
| Liu 2021b^92^ | **5** | 1 | 0 | 1 | 1 | 0 | 0 | 1 | 1 |
| Ma 2019^93^ | **6** | 1 | 0 | 1 | 1 | 0 | 1 | 1 | 1 |
| Manolakopoulos 2021^94^ | **5** | 0 | 0 | 1 | 1 | 0 | 1 | 1 | 1 |
| Nagata 2016^95^ | **5** | 1 | 0 | 1 | 1 | 0 | 1 | 0 | 1 |
| Ohlendorf 2022^96^ | **6** | 1 | 0 | 1 | 1 | 1 | 1 | 0 | 1 |
| Paik 2010^97^ | **6** | 1 | 1 | 1 | 1 | 0 | 1 | 1 | 0 |
| Pan 2015^98^ | **7** | 0 | 0 | 1 | 1 | 2 | 1 | 1 | 1 |
| Papatheodoridi 2017a^99^ | **3** | 0 | 0 | 0 | 1 | 0 | 0 | 1 | 1 |
| Papatheodoridis 2018a^100^ | **7** | 1 | 0 | 1 | 1 | 1 | 1 | 1 | 1 |
| Park 2016^101^ | **7** | 1 | 1 | 1 | 1 | 1 | 1 | 1 | 0 |
| Patwardhan 2014^102^ | **7** | 1 | 0 | 1 | 1 | 1 | 1 | 1 | 1 |
| Peng 2015^103^ | **3** | 0 | 0 | 1 | 1 | 0 | 0 | 1 | 0 |
| Peng 2021a^104^ | **6** | 0 | 0 | 1 | 1 | 1 | 1 | 1 | 1 |
| Peng 2021b^105^ | **5** | 1 | 1 | 1 | 1 | 0 | 0 | 1 | 0 |
| Petersen 2011a^106^ | **6** | 1 | 0 | 1 | 1 | 0 | 1 | 1 | 1 |
| Petersen 2013a^107^ | **6** | 1 | 0 | 1 | 1 | 0 | 1 | 1 | 1 |
| Pocurull 2022^108^ | **5** | 0 | 0 | 1 | 1 | 0 | 1 | 1 | 1 |
| Qiu 2016^109^ | **6** | 1 | 0 | 1 | 1 | 0 | 1 | 1 | 1 |
| Ridruejo 2014^110^ | **7** | 1 | 0 | 1 | 1 | 1 | 1 | 1 | 1 |
| Ryu 2003^111^ | **3** | 0 | 0 | 1 | 1 | 0 | 0 | 1 | 0 |
| Sah 2019a^112^ | **5** | 0 | 1 | 1 | 1 | O | 1 | 1 | 0 |
| Santos 2019^113^ | **6** | 1 | 0 | 1 | 1 | 0 | 1 | 1 | 1 |
| Sattayalertyanyong 2020a^114^ | **4** | 0 | 1 | 1 | 1 | 0 | 0 | 1 | 0 |
| Seto 2015^115^ | **5** | 1 | 1 | 1 | 1 | 0 | 0 | 0 | 1 |
| Seto 2016^116^ | **7** | 1 | 1 | 1 | 1 | 0 | 1 | 1 | 1 |
| Seto 2021^117^ | **6** | 1 | 0 | 1 | 1 | 0 | 1 | 1 | 1 |
| Shin 2005^118^ | **5** | 0 | 0 | 1 | 1 | 2 | 1 | 0 | 0 |
| Sohn 2014^119^ | **8** | 1 | 0 | 1 | 1 | 2 | 1 | 1 | 1 |
| Song 2012^120^ | **6** | 1 | 0 | 1 | 1 | 0 | 1 | 1 | 1 |
| Song 2018a^121^ | **3** | 0 | 1 | 1 | 1 | 0 | 0 | 0 | 0 |
| Song 2021^122^ | **3** | 0 | 0 | 1 | 1 | 0 | 0 | 1 | 0 |
| Sonneveld 2022a^123^ | **7** | 1 | 0 | 1 | 1 | 2 | 1 | 0 | 1 |
| Sonneveld 2022b^124^ | **5** | 1 | 0 | 1 | 1 | 0 | 1 | 0 | 1 |
| Su 2018^125^ | **8** | 1 | 1 | 1 | 1 | 1 | 1 | 1 | 1 |
| Su 2020^126^ | **7** | 1 | 1 | 1 | 1 | 2 | 0 | 1 | 0 |
| Su 2021^127^ | **3** | 0 | 0 | 1 | 1 | 1 | 0 | 0 | 0 |
| Suh 2012^128^ | **4** | 1 | 0 | 0 | 1 | 0 | 1 | 1 | 0 |
| Tseng 2018^129^ | **6** | 1 | 1 | 1 | 1 | 1 | 0 | 1 | 0 |
| Tseng 2020^130^ | **7** | 1 | 0 | 1 | 1 | 1 | 1 | 1 | 1 |
| Tseng 2022^131^ | **7** | 0 | 1 | 1 | 1 | 1 | 1 | 1 | 1 |
| Tsuge 2013^132^ | **5** | 1 | 0 | 1 | 1 | 0 | 1 | 0 | 1 |
| Tuefferd 2020^133^ | **3** | 0 | 0 | 1 | 1 | 0 | 0 | 1 | 0 |
| Tzu-Ning 2021^134^ | **4** | 0 | 0 | 1 | 1 | 0 | 0 | 1 | 1 |
| Wang 2010^135^ | **8** | 1 | 1 | 1 | 1 | 1 | 1 | 1 | 1 |
| Wang 2020^136^ | **4** | 0 | 0 | 1 | 1 | 0 | 0 | 1 | 1 |
| Wang 2022^137^ | **6** | 0 | 0 | 1 | 1 | 2 | 1 | 1 | 0 |
| Wong 2008^138^ | **8** | 1 | 1 | 1 | 1 | 2 | 1 | 1 | 0 |
| Xie 2022^139^ | **5** | 1 | 0 | 1 | 1 | 0 | 1 | 1 | 0 |
| Xu 2021^140^ | **6** | 1 | 1 | 1 | 1 | 0 | 0 | 1 | 1 |
| Xu 2022^141^ | **4** | 0 | 0 | 1 | 1 | 0 | 1 | 1 | 0 |
| Yao 2017^142^ | **7** | 0 | 0 | 1 | 1 | 2 | 1 | 1 | 1 |
| Yen 2018^143^ | **5** | 0 | 0 | 1 | 1 | 2 | 1 | 0 | 0 |
| Yoon 2005^144^ | **6** | 0 | 0 | 1 | 1 | 2 | 1 | 1 | 0 |
| Zhang 2017^145^ | **3** | 0 | 0 | 1 | 1 | 1 | 0 | 0 | 0 |

NOS: Newcastle-Ottawa Scale; *1 star=Truly representative of average target population in the community OR somewhat representative of average target population in the community, 0 star=Selected group of users e.g. nurses, volunteers, OR no description of the derivation of the cohort; **1 star=Drawn from the same community as the exposed cohort, 0 star=Drawn from a different source OR no description of the derivation; ***1star=Secure record (e.g. surgical records) or structured interview, 0 star=Written self-report or no description; ^#^1 star=Yes, 0 star=No; ^$^1 star=Study controls for most important confounder/covariate, 1 star=Study controls for additional confounders/covariates; ^1star=Independent blind assessment or record linkage, 0 star=Self-report or no description; ^$$^1 star=Yes (follow-up period of >1 year), 0 star=No (follow-up period of ≤1 year); ^^1 star=Complete follow-up - all subjects accounted for OR subjects lost to follow-up unlikely to introduce bias; i.e. small numbers (follow-up ≥70%) or description provided of those lost, 0 star=Follow-up rate too low (<70%) and no description of those lost OR no statement.

## Supplementary Table 5. Summary of participant characteristics

|  | **Number of studies reporting** | **Mean (number of studies)** | **Median (number of studies)** | **Proportion, %** |
| --- | --- | --- | --- | --- |
| **Age, years** | 59* | 27.8–60.3 (36) | 28.0–63.0 (26) |  |
| **Gender with any NA treatment, male** | 100 |  |  | 35–100 |
| **Baseline ALT** | 40* | 21–425.7 IU/mL (13) | 18–44 IU/mL (18) |  |
| **HBeAg status at NA start, negative**  **[in all patients] ^†^** | 31 |  |  | 27.8–98.1 |
| **HBeAg status at NA cessation, negative**  **[in patients with mixed HbeAg status at NA cessation] ^†^** | 5 |  |  | 33.1–92.3 |
| **HBsAg level at NA cessation** | 45* | 0.3–96.3 log IU/mL (25) | 0.3–233.2 log IU/mL (20) |  |
| **HBcrAg level at NA cessation** | 11* | 2.9–3.4 log IU/mL (3) | 2.5–4 log IU/mL (7) |  |
| **HBV DNA level** | 22* | Undetectable–<20 IU/mL (2)  Undetectable–7.4 log IU/mL (9)  5.2 log copies/mL (1) | 0–<20 IU/mL (4) Undetectable–5.5 log IU/mL (3) 2.7 μmol/L (1)  <0.7 mEq/mL (1)  3.5 log copies/mL (1) |  |
| **Cirrhosis, presence of** | 17 |  |  | 0–44.4 |
| **Duration of treatment with NA^‡^, weeks** | 85* | 36–597.5 weeks (41) | 24–571.6 weeks (46) |  |
| **Duration of total treatment with NA^§^, weeks** | 5* |  | 148–160 weeks (5) |  |

*As a continuous variable; ^†^Only 31/145 studies reported data on proportion of patients with HBeAg-negative status at NA start and 139/145 studies on HBeAg status at NA cessation (details are provided in Supplementary Table 5). Of these 139 studies, 113 studies included 100% patients with HBeAg-negative status at NA cessation, 6 studies included 100% patients with HBeAg-positive status at NA cessation and the remaining 20 studies included a mixed population of HBeAg-negative and HBeAg-positive patients. Among these 20 studies with a mixed HBeAg-status population, only five have provided further details of proportions data on patients with HBeAg-negative status at NA cessation (noted above in the present table); ^‡^Duration of the initial course of NA treatment; ^§^Total duration of treatment (initial course plus consolidation treatment).

ALT, alanine aminotransferase; AST, aspartate aminotransferase; HBcrAg, hepatitis B core-related antigen; HBeAg, hepatitis B e-antigen; HBsAg, hepatitis B surface antigen; HBV, hepatitis B virus; NA, nucleos(t)ide analogue.

## Supplementary Table 6. Number of studies reporting definitions for post NA cessation outcomes

| **Post NA cessation outcome** | **No. of studies reporting data for given outcome** | **No. of studies with specific definition of given outcome** | **No. of studies without any definition of given outcome** | **No. of clinical abstracts without any definition of given outcome** |
| --- | --- | --- | --- | --- |
| **Virological relapse** | 53 | 48 | 5 | 5 |
| **Clinical relapse** | 40 | 33 | 7 | 7 |
| **Biochemical relapse** | 10 | 10 | 0 | 0 |
| **Retreatment after discontinuation** | 14 | 12 | 2 | 1 |
| **HBsAg loss** | 24 | 3 | 21 | 4 |
| **HBsAg seroconversion** | 1 | 0 | 1 | 0 |
| **HBsAg seroreversion** | 1 | 0 | 1 | 0 |
| **HBeAg loss** | 1 | 1 | 0 | 0 |
| **HBeAg seroconversion** | 1 | 1 | 0 | 0 |
| **HBeAg seroreversion** | 7 | 6 | 1 | 0 |
| **Hepatic decompensation** | 5 | 3 | 2 | 1 |
| **Hepatic failure** | 1 | 1 | 0 | 0 |
| **Hepatic related death** | 3 | 3 | 0 | 0 |

HBsAg, hepatitis B surface antigen; HBeAg, hepatitis B e antigen; NA, nucleos(t)ide analogue.

## Supplementary Table 7. List of definitions for post NA cessation outcomes

| **Reported definitions (cumulative rates)** | **Number of studies** |
| --- | --- |
| **Virological relapse (n=53 studies)**^†^ | |
| Serum HBV DNA ≥2000 IU/mL in either a single or two distinct measurements, regardless of ALT level | 29 |
| Serum HBV DNA ≥10^4^ copies/mL in either a single or two distinct measurements | 9 |
| Serum HBV DNA detectable/reappearance/re-elevated or HBV DNA relapse | 3 |
| Serum HBV DNA ≥20,000 IU/mL in either a single or two distinct measurements | 2 |
| Serum HBV DNA >1000 copies/mL in either a single or two distinct measurements | 2 |
| Serum HBV DNA reappearance (cut-off value=1.4x10^5^ copies/mL) | 1 |
| Serum HBV DNA >200 IU/mL and any ALT | 1 |
| Serum HBV DNA >4.0 log copies/mL | 1 |
| Serum HBV DNA >60 IU/mL, regardless of biochemical response | 1 |
| Serum HBV DNA increase of ≥1 log copies/mL | 1 |
| Serum HBV DNA reappearance to a detectable level either by solution-hybridisation assay (lower limit of detection=2.83x10^5^ copies/mL) or  HBV DNA <10^5^ copies/mL by b-DNA method (Versant HBV DNA 3.0) | 1 |
| Definition NR | 5 |
| **Biochemical relapse (n=10 studies)**^†‡^ | |
| Serum ALT <2x ULN, serum ALT >2x ULN, serum ALT >5x ULN or serum ALT >10x ULN | 1 |
| Serum ALT >2x ULN | 3 |
| Serum ALT >10x ULN | 2 |
| Serum ALT >5x ULN | 1 |
| Serum ALT ≥5x or 10x ULN | 1 |
| Serum ALT ≥5x ULN with or without virological relapse | 1 |
| Serum ALT >ULN | 1 |
| Serum ALT >500 IU/L | 1 |
| Serum ALT abnormal (150 IU/L) after a period of ALT normalisation | 1 |
| Serum ALT >50 IU/L after NA discontinuation in patients whose ALT level had normalised (≤35 IU/L) at the end of NA therapy or  Serum ALT >80 IU/L (2x ULN) after NA discontinuation in patients whose ALT level was still high (>35 IU/L) at the end of NA therapy | 1 |
| **Clinical relapse (n=40 studies)**^†^ | |
| Serum HBV DNA ≥2000 IU/mL and serum ALT ≥2x ULN | 28 |
| HBV DNA >2000 IU/mL + ALT >1x ULN | 3 |
| HBV DNA >2000 IU/mL + ALT >1.25x ULN/doubling of ALT level from cessation with re-initiation of antiviral therapy | 1 |
| HBV DNA ≥2000 IU/mL + ALT ≥5x ULN | 1 |
| HBV DNA >2000 IU/mL + ALT >10x ULN | 1 |
| HBV DNA >2000 IU/mL + at least one of ALT or AST >2x ULN | 1 |
| HBV DNA >1x104 copies/mL from an undetectable level followed by ALT elevation >ULN | 1 |
| HBV DNA reappearance (by the Digen Hybrid Capture II assay) + serum transaminase at least 3x ULN | 1 |
| Definition NR | 7 |
| **Retreatment after discontinuation (n=14 studies)** | |
| Retreatment after NA discontinuation per Taiwan’s National Health Insurance Plan* | 4 |
| High ALT level and HBV DNA level, sufficiently elevated during the follow-up to necessitate retreatment with NA, to avoid fulminant hepatitis or cirrhosis | 1 |
| HBeAg-negative decompensated patients with total bilirubin ≥2 mg/dL or prolonged prothrombin time ≥3 seconds, regardless of HBV DNA level | 1 |
| Based on treating physician’s decision | 1 |
| Previously used NA (ETV or TDF) was used for retreatment as appropriate | 1 |
| Per varying retreatment criteria in the same study:  ALT>10x ULN, ALT>5x ULN and total bilirubin >2mg/dl at the same visit or  ALT>3x ULN and HBV DNA >100,000 IU/mL at the same visit and ALT >ULN and HBV DNA >2000 IU/mL on three sequential visits or  according to patients and physicians’ decisions in case of virological relapse with HBV DNA >20,000 IU/mL | 1 |
| Per varying retreatment criteria in the same study  Cirrhosis with detectable HBV DNA OR non-cirrhosis with clinical relapse prolonged over 3 months or  clinical relapse with serum bilirubin level >2 mg/dL or INR >1.5 OR based on joint physician-patient discussion | 1 |
| Per varying retreatment criteria in the same study:  Two consecutive ALT measurements >10x ULN regardless of the HBV DNA level or  ALT >5–10 ULN and HBV DNA >2,000 IU/mL persisting for ≥28 days (4 weeks) or  ALT >2–5 ULN and HBV DNA >2,000 IU/mL persisting for ≥6 months or  need for immunosuppressive treatment | 1 |
| Per varying retreatment criteria in different countries in the same study** | 1 |
| Definition NR | 2 |
| **HBsAg-related outcomes** | |
| **HBsAg loss (n=24 studies)** | |
| HBsAg loss defined in terms of functional cure | 1 |
| Undetectable HBsAg at any time during off-treatment follow-up | 1 |
| HBsAg loss after NA cessation with or without seroconversion to hepatitis B surface antibody (anti-HBs) and HBsAg loss after biochemical relapse | 1 |
| Definition NR | 21 |
| **HBsAg seroconversion (n=1 study)** | |
| Definition NR | 1 |
| **HBsAg seroreversion (n=1 study)** |  |
| Definition NR | 1 |
| **HBeAg-related outcomes** | |
| **HBeAg loss (n=1 study)** | |
| HBeAg clearance alone upon discontinuation of LAM (post-treatment durability) | 1 |
| **HBeAg seroconversion (n=1 study)** | |
| Seronegative conversion of HBeAg and HBV DNA associated with anti-HBe seropositivity on two consecutive occasions at least 2 months apart upon discontinuation of LAM | 1 |
| **HBeAg seroreversion (n=7 studies)** | |
| HBeAg-positivity/reappearance after prior HBeAg seroconversion (serologic recurrence) | 2 |
| HBeAg reappearance during follow-up period after treatment cessation | 2 |
| HBeAg reappearance at two consecutive protocol-defined visits | 1 |
| HBeAg reversion along with HBV DNA detectable but <2000 IU/mL and without ALT elevation | 1 |
| Definition NR | 1 |
| **Other clinical outcomes** | |
| **Hepatic decompensation (n=5 studies)**^†^ | |
| Severe clinical syndrome with hepatic function impairment as indicated by jaundice and a prolonged prothrombin time and/or occurrence of ascites/encephalopathy in patients with or without cirrhosis | 1 |
| Total bilirubin level >3 mg/dL, or prolonged prothrombin time >18 seconds after discontinuation of NA therapy | 1 |
| Severe acute exacerbation (SAE): SAE was defined as hepatitis B flare (HBV viral load >2000 IU/mL and ALT >5x ULN) with jaundice (total bilirubin ≥2 mg/dL), and/or coagulopathy (prolonged prothrombin time ≥3 seconds) | 1 |
| HD was reported if NA users were admitted or enrolled in the Registry for Catastrophic Illness Patient Database (RCIPD) with one of the following diagnoses: ascites (ICD-9 code: 789.5), hepatic encephalopathy (ICD-9 code: 572.2), portal hypertension (ICD-9 code: 572.3), hepatorenal syndrome (ICD-9 code: 572.4), or oesophageal or gastric varices (ICD-9 codes: 456.0, 456.1, 456.2, and 456.8) | 1 |
| Definition NR | 2 |
| **Hepatic failure (n=1 study)** | |
| Hepatic failure was defined if patients were admitted or enrolled in the Registry for Catastrophic Illness Patient Database with a diagnosis of hepatic failure (ICD-9 code: 570) or received liver transplantation | 1 |
| **Hepatic-related death (n=3 studies)** | |
| Death due to HD in patients with cirrhosis | 1 |
| Liver-related mortality due to cirrhosis, HCC, or HD | 1 |
| Death within 6 months following severe hepatitis flare | 1 |

*Non-cirrhotic HBeAg-positive: HBeAg reversion + HBV DNA ≥20,000 IU/mL + ALT >5X ULN OR ALT ≥2x ULN obtained (two measurements, 3 months apart), Non-cirrhotic HBeAg-negative: HBV DNA ≥2000 IU/mL + ALT ≥2x ULN obtained (two measurements, 3 months apart), Patients with HD: Total bilirubin ≥2 mg/dL or prolonged prothrombin time ≥3 seconds, regardless of HBV DNA level; **At the discretion of the treating physician (Belgium, Germany, Spain), Virologic relapse, combined relapse, ALT >10x ULN, ALT >3x ULN, and HBV DNA >100,000 IU/mL at the same visit, ALT >ULN and HBV DNA >2000 IU/mL on three sequential visits, patients’ and physicians’ decisions in case of HBV DNA >20,000 IU/mL (Greece), At the discretion of the treating physician or patient’s own initiative (Netherlands), Virologic relapse regardless of ALT level (Hong Kong), Per Taiwan’s national health plan, hyperbilirubinemia (serum total bilirubin >2 mg/dL), coagulopathy (prolonged prothrombin time >3 seconds), combined relapse, at the discretion of the treating physician, patient’s own initiative (Taiwan), HBeAg seroreversion, HBV DNA >2000 IU/mL and ALT >600 IU/mL at any visit, HBV DNA >2000 IU/mL and ALT >5x ULN on two consecutive visits, HBV DNA >2000 IU/mL and ALT >200 IU/mL but <600 IU/mL for >6–8 weeks, HBV DNA >20,000 IU/mL on two consecutive visits at least 4 weeks apart, at the discretion of the treating physician (Canada); †The sum of studies may exceed the number of studies following each definition as few studies followed more than one reporting definition for the same post NA cessation outcome. ^‡^ALT ULN was undefined in 6/7 studies; ALT ULN was defined as 40 U/L for males and 30 U/L for females in Chi 2015a.
ALT, alanine aminotransferase; anti-HBs, antibodies of Hepatitis B surface antigen; ETV, entecavir; HBsAg, hepatitis B surface antigen; HBeAg, hepatitis B e antigen; HBV DNA, hepatitis B virus deoxyribonucleic acid; HD, hepatic decompensation; HCC, hepatocellular carcinoma; ICD, International Classification of Diseases; LAM, lamivudine; NA, nucleos(t)ide analogue; NR, not reported; RCIPD, Registry for Catastrophic Illness Patient Database; SAE, severe acute exacerbation; TDF, tenofovir disoproxil fumarate; ULN, upper limit of normal.

## Supplementary Table 8. Overview of virological relapse rates

| **Virological relapse rates (range, median*) at key timepoints post NA cessation** | | | | | | | |
| --- | --- | --- | --- | --- | --- | --- | --- |
| **Study group** | **No. of studies** | **24 weeks** | **48 weeks** | **96 weeks** | **144 weeks** | **192 weeks** | **240 weeks** |
| **NA-D (any)** | 28 | 0.0–75.0%, median: 34.5 (n=14) | 0.0–91.7%, median: 51.5 (n=24) | 0.0–90.0%, median: 61 (n=14) | 23.4–94.0%, median: 57.7 (n=10) | 59.7–85.0% (n=2) | - |
| **ADV-D** | 1 | 13.5% (n=1) | - | 25.4% (n=1) | - | - | - |
| **ETV-D** | 17 | 9.1–74.2%, median: 21.75 (n=13) | 5.4–93.2%, median: 53.5 (n=15) | 40.4–96.0%, median: 61 (n=10) | 4.5–100.0%, median: 64.3 (n=5) | 80.0% (n=1) | - |
| **LAM-D** | 11 | 58.0–60.5% (n=2) | 13.6–66.0%, median: 36.6 (n=7) | 20.8–70.8%, median: 53.2 (n=6) | 4.0–87.8%, median: 36.7 (n=4) | - | - |
| **LdT-D** | 2 | 36.3% (n=1) | 9.6–66.9% (n=1) | 14.4–67.2% (n=2) | 38.3–67.5% (n=1) | - | - |
| **TDF-D** | 8 | 36.1–65.3%, median: 55.3 (n=7) | 20.4–80.4%, median: 65.6 (n=7) | 21.9–85.7%, median: 72.4 (n=5) | 13.8–87.5%, median: 72 (n=4) | 80.0% (n=1) | - |
| **(ADV+LAM)-D** | 1 | 59.9% (n=1) | - | 70.5%  (n=1) | - | - | - |
| **HBeAg-**  **(NA start)** | 14 | 12.8–75.0%, median: 33 (n=9) | 10.0–79.0%, median: 46.75 (n=13) | 41.7–80.0%, median: 70 (n=8) | 49.0–83.0%, median: 69.2 (n=5) | 85.0% (n=1) | - |
| **HBeAg+**  **(NA start)** | 11 | 10.8–60.0%, median: 34 (n=9) | 9.2–70.0%, median: 40.6 (n=11) | 41.3–72.4%, median: 54.2 (n=7) | 23.4–74.0%, median: 53 (n=4) | 72.0% (n=1) | - |

*Median values are not provided if ≤2 studies contributed to the outcome; ADV, adefovir dipivoxil; -D, discontinued; ETV, entecavir; HBeAg-, hepatitis B e-antigen negative; HBeAg+, hepatitis B
e-antigen positive; LAM, lamivudine; LdT, telbivudine; NA, nucleos(t)ide analogue; n, number of studies; TDF, tenofovir disoproxil fumarate.

## Supplementary Table 9. Overview of biochemical relapse rates

| **Biochemical relapse rates (range, median*) at key timepoints post NA cessation** | | | | | | | |
| --- | --- | --- | --- | --- | --- | --- | --- |
| **Study group** | **No. of studies** | **24 weeks** | **48 weeks** | **96 weeks** | **144 weeks** | **192 weeks** | **240 weeks** |
| **Clinical trials** | | | | | | | |
| **NA-D (any)** | 1 | 11.1–48.9% (n=1) | 13.3–40.0% (n=1) | - | - | - | - |
| **Observational studies** | | | | | | | |
| **NA-D** | 5 | 5.0–63.0%, median: 22.15 (n=3) | 3.2–71.1%, median: 21.3 (n=5) | 4.5–69.0%, median: 25 (n=3) | 5.9–73.0%, median: 29.7 (n=3) | 25.0–60.0% (n=1) | - |
| **LAM-D** | 3 | 48.0% (n=1) | 64.8% (n=1) | 69.2% (n=1) | 0.0–69.2% (n=2) | - | - |
| **HBeAg- (NA start)** | 1 | 10.0% (n=1) | 18.0% (n=1) | 25.0% (n=1) | 29.0% (n=1) | 30.0% (n=1) | - |
| **HBeAg+ (NA start)** | 1 | 5.0% (n=1) | 18.0% (n=1) | 25.0% (n=1) | 29.7% (n=1) | 30.0% (n=1) | - |

*Median values are not provided if ≤2 studies contributed to the outcome; ADV, adefovir dipivoxil; -D, discontinued; ETV, entecavir; HBV, hepatitis B virus; LAM, lamivudine; LdT, telbivudine; NA, nucleos(t)ide analogues; TDF, tenofovir disoproxil fumarate.

## Supplementary Table 10. Overview of retreatment after discontinuation rates

| **Retreatment after discontinuation rates (range, median*) at key timepoints post NA cessation** | | | | | | | |
| --- | --- | --- | --- | --- | --- | --- | --- |
| **Study group** | **No. of studies** | **24 weeks** | **48 weeks** | **96 weeks** | **144 weeks** | **192 weeks** | **240 weeks** |
| **Observational studies** | | | | | | | |
| **NA-D (any)** | 11 | 7.4–30.0%, median: 16.2 (n=5) | 11.4–40.0%, median: 28.1 (n=10) | 17–65.0%, median: 36.5 (n=8) | 23.4–77.7%, median: 46 (n=6) | 23.4–58.6% (n=1) | - |
| **ETV-D** | 3 | 0.0–18.1%, median: 1.7 (n=3) | 15.8–32.1%, median: 15.9 (n=3) | 23.9–48.3%, median: 36.8 (n=3) | 29.4–53.4%, median: 45.25 (n=3) | 54.3% (n=1) | - |
| **TDF-D** | 5 | 11.6–18.1%, median: 13 (n=5) | 25.8–41.1%, median: 27.1 (n=5) | 35.8–49.0%, median: 40.85 (n=4) | 44.3–55.4%, median: 46.8 (n=4) | 51.2% (n=1) | - |
| **HBeAg-  (NA start)** | 5 | 1.6–25.0%, median: 12.1 (n=4) | 15.8–39.1%, median: 25.9 (n=5) | 28.0–43.1%, median: 36.65 (n=4) | 44.3–53.1%, median: 45.65 (n=3) | 54.0% (n=1) | - |
| **HBeAg+ (NA start)** | 4 | 0.0–16.1%, median: 13 (n=3) | 15.8–41.1%, median: 23.4 (n=4) | 23.9–49.0%, median: 38.2 (n=3) | 29.4–55.4% (n=2) | 44.0% (n=1) | - |

*Median values are not provided if ≤2 studies contributed to the outcome; ADV, adefovir dipivoxil; -D, discontinued; ETV, entecavir; HBeAg-, hepatitis B e-antigen negative; HBeAg+, hepatitis B e-antigen positive; LAM, lamivudine; LdT, telbivudine; NA, nucleos(t)ide analogue; n, number of studies; TDF, tenofovir disoproxil fumarate.

## Supplementary Table 11. Overview of HBeAg-related outcomes

| **HBeAg-related outcome rates (range, median*) at timepoints of interest post NA cessation** | | | | | | | | | | |
| --- | --- | --- | --- | --- | --- | --- | --- | --- | --- | --- |
| **Study group** | **No. of studies** | **24 weeks** | **48 weeks** | **96 weeks** | **144 weeks** | **192 weeks** | **216 weeks** | **240 weeks** | **260 weeks** | **520 weeks** |
| **Observational studies – HBeAg loss** | | | | | | | | | | |
| **LAM-D** | 1 | - | - | - | - | - | - | - | 68.0% (n=1) | - |
| **Observational studies – HBeAg seroconversion** | | | | | | | | | | |
| **LAM-D** | 1 | 2.9% (n=1) | 20.0% (n=1) | 28.6% (n=1) | - | - | - | - | - | - |
| **Observational studies – HBeAg seroreversion** | | | | | | | | | | |
| **NA-D (any)** | 4 | 0% (n=1) | 7.0–12.1%, median: 8.6 (n=3) | 11.5–12.1% (n=2) | 12.1% (n=1) | 12.1% (n=1) | 16.2% (n=1) | - | - | - |
| **ETV-D** | 1 | 6.1–6.3% (n=1) | 20.2–23.1% (n=1) | 30.0–38.0% (n=1) | - | - |  | - | - | - |
| **LAM-D** | 2 | - | 33.3% (n=1) | - | 21.3–23.04% (n=1) | - | - | - | 9.0% (n=1) | - |
| **LdT-D** | 1 | - | 0.0–37.1% (n=1) | 0.0–37.1% (n=1) | 0.0–22.1% (n=1) | - | 0.0–22.1% (n=1) | - | - | - |
| **TDF-D** | 1 | 23.6–27.1% (n=1) | 29.7–37.5% (n=1) | 28.0–30.0% (n=1) | - | - | - | - | - | - |

*Median values are not provided if ≤2 studies contributed to the outcome; ADV, adefovir dipivoxil; -D, discontinued; ETV, entecavir; HBeAg-, hepatitis B e-antigen negative; HBeAg+, hepatitis B e-antigen positive; LAM, lamivudine; LdT, telbivudine; NA, nucleos(t)ide analogue; n, number of studies; TDF, tenofovir disoproxil fumarate.

## Supplementary Table 12. Summary of other clinical outcome rates following NA cessation

| **Hepatic-related death rates (range, median*) at timepoints of interest** | | | | | | | | | | |
| --- | --- | --- | --- | --- | --- | --- | --- | --- | --- | --- |
| **Study group** | **No. of studies** | **24 weeks** | **48 weeks** | **96 weeks** | **144 weeks** | **192 weeks** | **216 weeks** | **240 weeks** | **260 weeks** | **520 weeks** |
| **Observational studies – hepatic decompensation** | | | | | | | | | | |
| **NA-D (any)** | 4 | 0.0–17.0% (n=1) | 0.0–23.0%, median: 3.1 (n=5) | 1.3–15.4% (n=1) | 1.04–17.42% (n=1) | - | 1.3–17.1% (n=1) | - | 0.0–25.91% median: 1.04 (n=3) | - |
| **LAM-D** | 1 | - | 8.2% (n=1) | 12.5% (n=1) | - | - | - | - | 18.1–21.2%  (n=1) | - |
| **HBeAg- (NA start)** | 1 | - | 0.3% (n=1) | - | - | - |  |  |  |  |
| **Observational studies – hepatic failure** | | | | | | | | | | |
| **NA-D (any)** | 1 | - | 0.4% (n=1) | - | - | - | - | - | - | - |
| **Observational studies – hepatic-related death** | | | | | | | | | | |
| **NA-D (any)** | 2 | - | 0.3% (n=1) | 0.4% (n=1) | 0.5% (n=1) | - | 0.5% (n=1) | - | 0.0–3.0% (n=1) | 0.0–6.0% (n=1) |
| **LAM-D** | 1 | - | 0.0% (n=1) | - | - | - | - | - | 0.0–2.9% (n=1) | - |

*Median values are not provided if ≤2 studies contributed to the outcome; ADV, adefovir dipivoxil; D, discontinued; ETV, entecavir; HBeAg, hepatitis B e-antigen negative; HBeAg+, hepatitis B e-antigen positive; LAM, lamivudine; LdT, telbivudine; NA, nucleos(t)ide analogue; TDF, tenofovir disoproxil fumarate.

## Supplementary Table 13. Summary of significant predictors of virological relapse following NA cessation

| Variable/factor | Available literature | Consistency of association | Summary finding (based on quantitative multivariate analysis) |
| --- | --- | --- | --- |
| **Demographic variables** | | | |
| Age | Adequate | Consistent | - ↑ age a/w ↑ risk of virological relapse (HR: 1.028-1.751) (n=9 studies); age <40 years (vs. >40 years) a/w ↓ risk of virological relapse (HR: 0.520, 95% CI: 0.264-0.998) (n=1 study) - ↑ age a/w ↑ risk of virological relapse (OR: 1.060-10.959) (n=5 studies); ↑ age a/w ↓ risk of virological relapse (OR: 0.404, 95% CI: 0.200-0.935 (n=1 study) - ↑ age a/w ↑ risk of virological relapse (≤40 years vs. >40 years) (RR: 0.310, 95% CI: 0.096-0.998) (n=1 study) - ↑ age a/w ↑ risk of virological relapse (Exp (B)/OR: 1.304, 95% CI: NR) (n=1 study) |
| Gender | Limited | Consistent | - Male gender a/w ↑ risk of virological relapse (vs. female gender) (HR: 2.040-2.354) (n=3 studies) |
| **Biochemical variables** | | | |
| ALT | Limited | Consistent | - ↑ ALT (BL/EOT) a/w ↑ risk of virological relapse (HR: 1.060, 95% CI: 1.010-1.110) (n=1 study) - ↑ ALT (BL/EOT) a/w ↑ risk of virological relapse (OR: 1.080, 95% CI: 1.010-1.160) (n=1 study) |
| Bilirubin | Limited | Consistent | - ↑ Total bilirubin (at NA cessation) a/w ↓ risk of virological relapse (HR: 0.092, 95% CI: 0.020-0.418) (n=1 study) - ↑ Total bilirubin ≥2 mg/dL (at NA start) a/w ↓ risk of virological relapse (HR: 0.621, 95% CI: 0.455-0.847) (n=1 study) |
| **Viral markers** | | | |
| HBsAg | Adequate | Consistent | - ↑ HBsAg (BL/EOT) a/w ↑ risk of virological relapse (HR: 1.007-8.930) (n=17 studies) - ↑ HBsAg (BL/EOT) a/w ↑ risk of virological relapse (OR: 2.000-2.734) (n=2 studies) - ↑ HBsAg (EOT) a/w ↓ risk of virological relapse (OR:0.812, 95% CI: NR) (n=1 study) - ↑ HBsAg (BL/EOT) a/w ↑ risk of virological relapse (RR: 2.041, 95% CI: 1.098-3.194) (n=1 study) |
| HBV DNA | Adequate | Consistent | - ↑ HBV DNA (BL) a/w ↑ risk of virological relapse (HR: 1.127-3.003) (n=9 studies) - ↑ HBV DNA (BL) a/w ↑ risk of virological relapse (OR: 3.500-13.333) (n=4 studies) - ↑ HBV DNA (BL) a/w ↑ risk of virological relapse (Exp (B): 12.485, 95% CI: NR) (n=1 study) |
| HBV RNA | Adequate | Consistent | - ↑ HBV RNA (EOT) a/w ↑ risk of virological relapse (HR: 2.278-3.790) (n=3 studies) - HBV RNA positivity a/w ↑ risk of virological relapse (OR: 14.590-14.590) (n=2 studies) |
| HBcrAg | Adequate | Consistent | - ↑ HBcrAg (EOT) a/w ↑ risk of virological relapse (OR: 3.751-14.140) (n=3 studies) - ↑ HBcrAg (EOT) a/w ↑ risk of virological relapse (HR: 2.430, 95% CI:1.907-5.385) (n=1 study) - ↑ HBcrAg (BL) a/w ↑of virological relapse (HR: 1.840, 95% CI: 1.417-2.388) (n=1 study) - ↑ HBcrAg (EOT) a/w ↓ risk of virological relapse (OR: 0.701, 95% CI: NR) (n=1 study) |
| HBeAg status | Limited | Inconsistent | - HBeAg-positivity (at NA start) a/w ↑ risk of virological relapse (HR: 1.900, 95% CI: NR) (n=1 study) - HBeAg-positivity (before NA start) a/w ↓ risk of virological relapse (HR: 0.470, 95% CI: 0.230-0.950) (n=1 study) - HBeAg-positivity (at NA start) a/w ↓ of virological relapse (OR: 0.320-0.400, 95% CI: 0.140-0.200, 0.740-0.800) (n=1 study) - HBeAg-positivity (EOT) a/w ↑ risk of virological relapse (OR: 26.667, 95% CI: 2.242-317.147) (n=1 study) |
| HBV genotype | Limited | Inconsistent | - HBV genotype C a/w ↑ risk of virological relapse (HR: 2.930, 95% CI: 1.380-6.230) (n=1 study) - HBV genotype C a/w ↓ of virological relapse (HR: 0.581, 95% CI: 0.411-0.820) (n=1 study) |
| HBV DNA + HBV RNA | Limited | Consistent | - ↑ HBV DNA + HBV RNA titres (at 3 months of NA Rx) a/w ↑ risk of virological relapse (OR: 9.474, 95% CI: 1.069-83.957) (n=1 study) - HBV DNA + HBV RNA positivity a/w ↑ risk of virological relapse (HR: 11.100 95% CI: 2.690-45.809) (n=1 study) |
| HBsAg + HBV DNA | Very limited | Consistent | - HBsAg ≥40 IU/mL (EOT) + HBV DNA of 5 x10^4^ IU/mL (BL) a/w ↑ risk of virological relapse (HR: 2.360, 95% CI: 1.750-3.170) (n=1 study) |
| HBsAg + HBcrAg | Very limited | Consistent | - HBsAg ≥40 IU/mL (EOT) + HBcrAg of 4 log U/mL (BL) a/w ↑ risk of virological relapse (HR: 2.450, 95% CI: 1.820-3.300) (n=1 study) |
| **Liver disease-related variables** | | | |
| Liver cirrhosis | Very limited | Consistent | - Liver cirrhosis (present vs. absent) a/w ↓ of virological relapse (HR: 0.190, 95% CI: 0.040-0.860) (n=1 study) |
| **NA treatment-related variables** | | | |
| Duration of NA consolidation | Adequate | Consistent | - Longer duration of NA consolidation a/w ↓ of virological relapse (HR: 0.180-0.958) (n=4 studies) - Longer duration of NA consolidation a/w ↓ of virological relapse (<15 months vs. ≥15 months) (RR: 11.299, 95% CI: 2.054-65.120) (n=1 study) - Longer duration of NA consolidation a/w ↑ risk of virological relapse (OR: 10.227, 95% CI: 1.051-1.432) (n=1 study) |
| Type of NA Rx-D | Adequate | Consistent | - TDF therapy (vs. ETV therapy) a/w ↑ risk of virological relapse (HR: 1.600-3.335) (n=3 studies) - TDF therapy (vs. ETV therapy) a/w ↑ risk of virological relapse (OR: 13.100, 95% CI: 2.180-79.300 (n=1 study) - TDF+LAM therapy a/w ↑ risk of virological relapse (OR: 5.300, 95% CI: 1.100-24.800) (n=1 study) |
| Prior NA exp. | Limited | Inconsistent | - Prior NA experience a/w ↓ of virological relapse (HR: 0.540, 95% CI: 0.310-0.950) (n=1 study) - Prior NA experience a/w ↑ risk of virological relapse (HR: 2.780, 95% CI: 1.150-6.710) (n=1 study) |
| Duration of NA Rx | Very limited | Consistent | - Longer duration of NA treatment a/w ↑ risk of virological relapse (HR: 1.400, 95% CI: 1.140-1.730) (n=1 study) |
| LAM resistance | Very limited | Consistent | - Lamivudine resistance (present vs. absent) a/w ↑ risk of virological relapse (HR: 3.650, 95% CI: 1.1781-7.482) (n=1 study) |
| **Other reported variables** | | | |
| Time to UD HBV DNA | Limited | Consistent | - Longer time to achieve undetectable HBV DNA a/w ↑ risk of virological relapse (HR: 1.246-1.292) (n=2 studies) - Longer time to achieve undetectable HBV DNA a/w ↑ risk of virological relapse (OR: 1.311, 95% CI: 1.014-1.694) (n=1 study) |
| SCALE-B score | Very limited | Consistent | - SCALE-B score ≥320 (highest strata) vs. 260 (lowest strata) a/w ↑ risk of virological relapse (HR: 5.000, 95% CI: 1.800-14.400) (n=1 study) |
| Time to HBeAg SC | Very limited | Consistent | - Longer time to achieve HBeAg seroconversion a/w ↑ risk of virological relapse (HR: 0.943, 95% CI: 0.898-0.990) (n=1 study) |
| Duration of neg. HBV DNA maintenance | Very limited | Consistent | - Longer duration of negative HBV DNA maintenance a/w ↓ of virological relapse (HR: 0.700, 95% CI: 0.660-1.000) (n=1 study) |
| Time to CR | Very limited | Consistent | - Longer course to CR (UD HBV DNA + HBeAg SC + normal ALT) a/w ↑ risk of virological relapse (HR: 1.038, 95% CI: 1.002-1.076) (n=1 study) |
| Time to CVR | Very limited | Consistent | - Longer time to CVR a/w ↑ risk of virological relapse (HR: 1.006, 95% CI: 1.003-1.009) (n=1 study) |

a/w, associated with; exp., experienced; neg, negative; ALT, alanine aminotransferase; BL, baseline; CI, confidence interval; CR, complete response; CVR, complete virological response; -D, discontinued; DNA, deoxynucleic acid; EOT, end of treatment; Ex(p) B, exponential B coefficient; HBcrAg, hepatitis B core-related antigen; HBeAg, hepatitis B e-antigen; HR, hazard ratio; HBsAg, hepatitis B surface antigen; HBV, hepatitis B virus; LAM, lamivudine; NA, nucleos(t)ide analogue; NR, not reported; RNA, ribonucleic acid; RR, risk ratio; OR, odds ratio; Rx, treatment; Rx-D, treatment discontinued; SC, seroconversion; TDF, tenofovir disoproxil fumarate; UD, undetectable.
Note: Available literature: Very limited (1 study only); Limited (2-4 studies); Adequate (≥5 studies). SCALE‐B score=35 ×HBsAg + 20 × HBcrAg + 2 × age + 40 for TDF use.

## References

1. Berg T, Simon KG, Mauss S, et al. Long-term response after stopping tenofovir disoproxil fumarate in non-cirrhotic HBeAg-negative patients - FINITE study. *J Hepatol.* 2017;67(5):918-924.

2. Brouwer WP, Xie Q, Sonneveld MJ, et al. Adding pegylated interferon to entecavir for hepatitis B e antigen-positive chronic hepatitis B: A multicenter randomized trial (ARES study). *Hepatology.* 2015;61(5):1512-1522.

3. Dienstag JL, Schiff ER, Mitchell M, et al. Extended lamivudine retreatment for chronic hepatitis B: maintenance of viral suppression after discontinuation of therapy. *Hepatology.* 1999;30(4):1082-1087.

4. Dienstag JL, Schiff ER, Wright TL, et al. Lamivudine as initial treatment for chronic hepatitis B in the United States. *N Engl J Med.* 1999;341(17):1256-1263.

5. Feld JJ, Wahed A, Ghany MG, et al. Treatment withdrawal after long-term nucleotide analogue therapy with or without peginterferon: predictors of alt flares, HBsAg decline and active disease. *Hepatology.* 2021;74:501a-502a.

6. Goulis I, Dalekos GN. Entecavir monotherapy for lamivudine-refractory chronic hepatitis B. *Expert Rev Anti Infect Ther.* 2008;6(6):855-859.

7. Johannessen A, Reikvam DH, Aleman S, et al. One-year safety results of the NUC-Stop Study, an open-label study on stopping antiviral therapy in HBeAg-negative chronic hepatitis B. *J Hepatol.* 2021;75:S734-S734.

8. Lai CL, Wong DK, Wong GT, Seto WK, Fung J, Yuen MF. Rebound of HBV DNA after cessation of nucleos/tide analogues in chronic hepatitis B patients with undetectable covalently closed. *JHEP Rep.* 2020;2(3):100112.

9. Liem KS, Fung S, Wong DK, et al. Limited sustained response after stopping nucleos(t)ide analogues in patients with chronic hepatitis B: results from a randomised controlled trial (Toronto STOP study). *Gut.* 2019;68(12):2206-2213.

10. Sung JJ, Wong ML, Bowden S, et al. Intrahepatic hepatitis B virus covalently closed circular DNA can be a predictor of sustained response to therapy. *Gastroenterology.* 2005;128(7):1890-1897.

11. Wong VW, Hui AJ, Wong GL, et al. Four-year outcomes after cessation of tenofovir in immune-tolerant chronic hepatitis B patients. *J Clin Gastroenterol.* 2018;52(4):347-352.

12. Zhang XQ, Zhang HY, You JP, Mao Q. Efficacy of pegylated interferon α2a in patients without HBeAg loss after the withdrawal of long-term lamivudine therapy. *Virol J.* 2013;10:21.

13. Abdurakhmanov D, Panevkina S, Ibragimov E, et al. End-of-treatment quantitative HBsAg <500 IU/mL can predict sustained remission and HBsAg loss after discontinuation of nucleos(t)ide analogues in chronic hepatitis B. The Liver Meeting Digital Experience™. AASLD 2020.

14. Akuta N, Suzuki F, Kobayashi M, et al. Virological and biochemical relapse after discontinuation of lamivudine monotherapy for chronic hepatitis B in Japan: comparison with breakthrough hepatitis during long-term treatment. *Intervirology.* 2005;48(2-3):174-182.

15. Alizadeh AHM, Ranjbar M, Karimi B, Hatami S. Biochemical response to lamivudine treatment in HBeAg negative chronic hepatitis B patients in Iran. *World J Gastroenterol.* 2006;12(26):4203-4205.

16. Assawasuwannakit S, Kaewdech, A, Sripongpun, P, et al. Clinical utility of Scale-B in the prediction of relapse and HBsAg loss after antiviral discontinuation in Asian chronic hepatitis B patients after 2 years follow-up. *Hepatology.* 2021;74:431a.

17. Azhari H, Frolkis AD, Shaheen AA, et al. Real world single centre experience on the efficacy of stopping long term nucleos(t)ide analog therapy in patients with chronic hepatitis. The Liver Meeting Digital Experience™. AASLD 2020.

18. Brakenhoff S, Robert J, Knegt D, et al. End of treatment HBsAg, HBcrAg and HBV RNA levels predict risk of off-treatment ALT flares in patients with cirrhotic hepatitis B. AASLD 2020. 72(1):114.

19. Broquetas T, Hernandez JJ, Garcia-Retortillo M, et al. On-treatment HBsAg kinetics can predict HBsAg loss after nucleos(t)ide analogues interruption in HBeAg-negative patients. EASL 2021. 75(2):S734-S734.

20. Buti M, Casillas R, Riveiro-Barciela M, et al. Tenofovir discontinuation after long-term viral suppression in HBeAg negative chronic hepatitis B. Can HBsAg levels be useful? *J Clin Virol.* 2015;68:61-68.

21. Byun KS, Kwon OS, Kim JH, et al. Factors related to post-treatment relapse in chronic hepatitis B patients who lost HBeAg after lamivudine therapy. *J Gastroenterol Hepatol.* 2005;20(12):1838-1842.

22. Chan HL, Wong GL, Chim AM, Chan HY, Chu SH, Wong VW. Prediction of off-treatment response to lamivudine by serum hepatitis B surface antigen quantification in hepatitis B e antigen-negative patients. *Antivir Ther.* 2011;16(8):1249-1257.

23. Chang ML, Jeng WJ, Liaw YF. Clinical events after cessation of lamivudine therapy in patients recovered from hepatitis B flare with hepatic decompensation. *Clin Gastroenterol Hepatol.* 2015;13(5):979-986.

24. Chaung KT, Ha NB, Trinh HN, et al. High frequency of recurrent viremia after hepatitis B e antigen seroconversion and consolidation therapy. *J Clin Gastroenterol.* 2012;46(10):865-870.

25. Chen CH, Lee CM, Hung C, et al. The role of hepatitis B surface antigen quantification predict HBV reactivation after discontinuation of entecavir treatment. *Hepatology.* 2013;58:655A-655A.

26. Chen L, Li X-L, Lin M-H, et al. Viral relapses develop more rapidly in HBeAg-negative chronic hepatitis B patients after nucleos(t)ide analogues withdraw. *Hepatol Int.* 2013;7:S199-S200.

27. Chen C-H, Lee C-M, Wang J-H, et al. The role of hepatitis B surface antigen quantification predict HBsAg loss and HBV reactivation after discontinuation of lamivudine treatment. *Hepatol Int.* 2013;7(1):S231-S231.

28. Chen DB, Chen YM, Liu J, et al. Durability of efficacy after telbivudine off-treatment in chronic hepatitis B patients. *J Clin Virol.* 2014;59(1):50-54.

29. Chen CH, Hung CH, Hu TH, et al. Association between level of hepatitis B surface antigen and relapse after entecavir therapy for chronic hepatitis B virus infection. *Clin Gastroenterol Hepatol.* 2015;13(11):1984-1992.e1981.

30. Chen CH, Lee CM, Hung CH, et al. The incidence and predictors of hepatitis B surface antigen loss and hepatocellular carcinoma development after the cessation of lamivudine and entecavir treatment in chronic hepatitis B patients. *J Gastroenterol Hepatol.* 2015;30:379-379.

31. Chen C-H, Hu T-H, Lu S-N, et al. Comparison of HBV relapse rates between patients who discontinue entecavir and tenofovir treatment in chronic hepatitis B patients. *Hepatol Int.* 2017;11(1):S697.

32. Chen CH, Hsu YC, Lu SN, et al. The incidence and predictors of HBV relapse after cessation of tenofovir therapy in chronic hepatitis B patients. *J Viral Hepat.* 2018;25(5):590-597.

33. Chen C, Wen R, Jeng J, et al. Comparison of hepatitis B virus relapses between hepatitis B e antigen-negative chronic hepatitis B patients who discontinue tenofovir disoproxil fumarate with or without switching to alafenamide. EASL 2021. 75(2):S747-S747.

34. Chen E, Wang F, Zhou J, et al. Serum levels of pgRNA and HBcrAg are associated with viral relapse after cessation of nucleotide analogues therapy in chronic hepatitis B patients. APASL 2022. 16(1):S71-S71.

35. Chi H, Hansen BE, Yim C, et al. Reduced risk of relapse after long-term nucleos(t)ide analogue consolidation therapy for chronic hepatitis B. *Aliment Pharmacol Ther.* 2015;41(9):867-876.

36. Chi H, Wong D, Peng J, et al. Durability of response after hepatitis B surface antigen seroclearance during nucleos(t)ide analogue treatment in a multiethnic cohort of chronic hepatitis b patients: results after treatment cessation. *Clin Infect Dis.* 2017;65(4):680-683.

37. Chi H, Li Z, Hansen BE, et al. Serum level of antibodies against hepatitis B core protein is associated with clinical relapse after discontinuation of nucleos(t)ide analogue therapy. *Clin Gastroenterol Hepatol.* 2019;17(1):182-191.e181.

38. Dienstag JL, Cianciara J, Karayalcin S, et al. Durability of serologic response after lamivudine treatment of chronic hepatitis B. *Hepatology.* 2003;37(4):748-755.

39. Fan R, Peng J, Xie Q, et al. Combining hepatitis B virus RNA and hepatitis B core-related antigen: guidance for safely stopping nucleos(t)ide analogues in hepatitis B e antigen-positive patients with chronic hepatitis B. *J Infect Dis.* 2020;222(4):611-618.

40. Fang HW, Yen YH, Hung CH, et al. Predictors of virological suppression after clinical relapse in patients who discontinued entecavir or tenofovir. *Dig Dis Sci.* 2022;67(7):3402-3411.

41. Fong TL, Tien A, Jo KJ, et al. Durability of hepatitis B e antigen seroconversion in chronic hepatitis B patients treated with entecavir or tenofovir. *Dig Dis Sci.* 2015;60(11):3465-3472.

42. Fung J, Lai CL, Tanaka Y, et al. The duration of lamivudine therapy for chronic hepatitis B: cessation vs. continuation of treatment after HBeAg seroconversion. *Am J Gastroenterol.* 2009;104(8):1940-1946; quiz 1947.

43. Gao L, Hu Y, Shi X, Li X, Zhang D, Ren H. 48 weeks outcome after cessation of nucleos(t)ide analogue therapy in chronic hepatitis B patients. *Ann Hepatol.* 2020;19(3):329-334.

44. Garcia-Lopez M, Lens S, Pallett LJ, et al. Serum and intrahepatic HBV markers and HBV-specific CD8 T cell responses after nucleos(t)ide analog therapy discontinuation in HBeAg-negative chronic hepatitis B patients. *J Hepatol.* 2020;73:S6.

45. Ge GH, Ye Y, Zhou XB, et al. Hepatitis B surface antigen levels of cessation of nucleos(t)ide analogs associated with virological relapse in hepatitis B surface antigen-negative chronic hepatitis B patients. *World J Gastroenterol.* 2015;21(28):8653-8659.

46. Guerra AF, Tomassoli G, Piermatteo L, et al. Serum HBsAg and ddPCR HBV-DNA as predictive parameters of HBsAg loss after nucleos(t)ide analogue (NA) treatment discontinuation in non-cirrhotic patients with chronic hepatitis B. EASL 2022. 77(SI):S839-S839.

47. He D, Guo S, Chen W, et al. Long-term outcomes after nucleos(t)ide analogues discontinuation in chronic hepatitis B patients with HBeAg-negative. *BMC Infect Dis.* 2013;13:458.

48. He D, Guo S, Zhu P, et al. Long-term outcomes after nucleos(t)ide analogue discontinuation in HBeAg-positive chronic hepatitis B patients. *Clin Microbiol Infect.* 2014;20(10):O687-693.

49. Hirode G, Choi HSJ, Chen CH, et al. Off-therapy response after nucleos(t)ide analogue withdrawal in patients with chronic hepatitis B: an international, multicenter, multiethnic cohort (RETRACT-B study). *Gastroenterology.* 2022;162(3):757-771.e754.

50. Hoener Zu Siederdissen C, Rinker F, et al. Interruption of nucleos(t)ide analogue therapy for HBeAg-negative chronic hepatitis B-a new concept to achieve HBsAg decline? *J Hepatol.* 2015;62:S571-S572.

51. Honkoop P, de Man RA, Niesters HG, Zondervan PE, Schalm SW. Acute exacerbation of chronic hepatitis B virus infection after withdrawal of lamivudine therapy. *Hepatology.* 2000;32(3):635-639.

52. Hsu YC, Nguyen MH, Mou LJ, et al. Hepatitis B core-related antigen and the novel scale-B score to predict relapse risk after cessation of nucleos(t)ide analogues in patients with chronic hepatitis B. *Hepatology.* 2018;68:236A-237A.

53. Hsu YC, Lin YH, Lee TY, et al. Severe hepatitis flare and related mortality after discontinuation of oral antiviral treatment in patients with chronic hepatitis B: a population-based study. *Hepatology.* 2021;74:18A-18A.

54. Huang YH, Lee I, Sun CK, et al. Durability of nucleos(t)ide analogues treatment in patients with chronic hepatitis B: The role of APASL guideline. *Hepatology.* 2014;60:1122A-1122A.

55. Hung CH, Wang JH, Lu SN, Hu TH, Lee CM, Chen CH. Hepatitis B surface antigen loss and clinical outcomes between HBeAg-negative cirrhosis patients who discontinued or continued nucleoside analogue therapy. *J Viral Hepat.* 2017;24(7):599-607.

56. Ito K, Tanaka Y, Orito E, et al. Predicting relapse after cessation of lamivudine monotherapy for chronic hepatitis B virus infection. *Clin Infect Dis.* 2004;38(4):490-495.

57. Jackson K, Visvanathan K, Hall S, et al. Serum HBV RNA levels are associated with risk of hepatitis flare after stopping NA therapy in HBeAg-negative patients. *J Hepatol.* 2022;77:S842.

58. Jang JW, Choi JY, Bae SH, et al. Stopping lamivudine therapy after biochemical breakthrough may be a feasible option in selected HBeAg-positive patients. *J Med Virol.* 2005;77(3):367-373.

59. Jeng WJ, Sheen IS, Chen YC, et al. Off-therapy durability of response to entecavir therapy in hepatitis B e antigen-negative chronic hepatitis B patients. *Hepatology.* 2013;58(6):1888-1896.

60. Jeng RWJ, Chen YC, Sheen IS, et al. Clinical relapse after cessation of tenofovir in HBeAg-negative patients: Presentation and predictor. *Hepatol Int.* 2016;10(1):S52.

61. Jeng WJ, Chen YC, Chien RN, Sheen IS, Liaw YF. Incidence and predictors of hepatitis B surface antigen seroclearance after cessation of nucleos(t)ide analogue therapy in hepatitis B e antigen-negative chronic hepatitis B. *Hepatology.* 2018;68(2):425-434.

62. Jeng RWJ, Liu YC, Peng CW, et al. HCC incidence is low after HBsAg seroclearance in off-NUC patients. *J Hepatol.* 2021;75:S749.

63. Jeng WJ, Chen CH, Liu YC, et al. Cirrhosis is the only independent factor for hepatocellular carcinoma in off-NUC patients with HBsAg seroclearance. *Hepatology.* 2021;74:472A-473A.

64. Jeng RWJ, Liu YC, Peng CW, et al. Long-term outcomes after cessation of antiviral therapy in HBeAg-negative patients. *J Hepatol.* 2022;77:S873.

65. Jiang JN, Huang ZL, He LX, et al. Residual amount of HBV DNA in serum is related to relapse in chronic hepatitis B patients after cessation of nucleos(t)ide analogs. *J Clin Gastroenterol.* 2015;49(4):323-328.

66. Jin YJ, Kim KM, Yoo DJ, et al. Clinical course of chronic hepatitis B patients who were off-treated after lamivudine treatment: analysis of 138 consecutive patients. *Virol J.* 2012;9:239.

67. Jun BG, Lee SH, Kim HS, et al. Predictive factors for sustained remission after discontinuation of antiviral therapy in patients with HBeAg-positive chronic hepatitis B. *Korean J Gastroenterol.* 2016;67(1):28-34.

68. Jung YK, Yeon JE, Lee KG, et al. Clinical outcomes after adefovir discontinuation in lamivudine-resistant chronic hepatitis B patients. *Hepatology.* 2009;50(4):529A-529A.

69. Jung YK, Yeon JE, Lee KG, et al. Virologic response is not durable after adefovir discontinuation in lamivudine-resistant chronic hepatitis B patients. *Korean J Hepatol.* 2011;17(4):261-267.

70. Jung KS, Park JY, Chon YE, et al. Clinical outcomes and predictors for relapse after cessation of oral antiviral treatment in chronic hepatitis B patients. *J Gastroenterol.* 2016;51(8):830-839.

71. Kaewdech A, Tangkijvanich P, Sripongpun P, et al. Hepatitis B surface antigen, core-related antigen and HBV RNA: Predicting clinical relapse after NA therapy discontinuation. *Liver Int.* 2020;40(12):2961-2971.

72. Kang SH, Kang K, Jong Eun Y, et al. Antiviral response is not sustained after cessation of lamivudine treatment in chronic hepatitis B patients: A 10-year follow-up study. *J Med Virol.* 2017;89(5):849-856.

73. Karakaya F, Özer S, Kalkan Ç, et al. Discontinuation of lamivudine treatment in HBeAg-negative chronic hepatitis B: a pilot study with long-term follow-up. *Antivir Ther.* 2017;22(7):559-570.

74. Kim JH, Lee SJ, Joo MK, et al. Durability of antiviral response in HBeAg-positive chronic hepatitis B patients who maintained virologic response for one year after lamivudine discontinuation. *Dig Dis Sci.* 2009;54(7):1572-1577.

75. Kim SS, Kim YJ, Ahn SJ, et al. Durability after discontinuation of nucleos(t)ide therapy in hepatitis e antigen negative chronic hepatitis B patients. *Hepatol Int.* 2013;7:S221.

76. Kim MA, Kim SU, Sinn DH, et al. Discontinuation of nucleos(t)ide analogues is not associated with a higher risk of HBsAg seroreversion after antiviral-induced HBsAg seroclearance: a nationwide multicentre study. *Gut.* 2020;69(12):2214-2222.

77. Kranidioti H, Manolakopoulos S, Kontos G, et al. Immunological biomarkers as indicators for outcome after discontinuation of nucleos(t)ide analogue therapy in patients with HBeAg-negative chronic hepatitis B. *J Viral Hepat.* 2019;26(6):697-709.

78. Kuo MT, Hu TH, Hung CH, et al. Hepatitis B virus relapse rates in chronic hepatitis B patients who discontinue either entecavir or tenofovir. *Aliment Pharmacol Ther.* 2019;49(2):218-228.

79. Kuo YH, Wang JH, Hung CH, Lu SN, Hu TH, Chen CH. Combining end-of-treatment HBsAg and baseline hepatitis B core-related antigen reduce HBV relapse rate after tenofovir cessation. *Hepatol Int.* 2021;15(2):301-309.

80. Lai CY, Yang SS, Lee SW, Tsai HJ, Lee TY. Cessation of nucleos(t)ide analogue therapy in non-cirrhotic hepatitis B patients with prior severe acute exacerbation. *J Clin Med.* 2021;10(21).

81. Lee KM, Cho SW, Kim SW, Kim HJ, Hahm KB, Kim JH. Effect of virological response on post-treatment durability of lamivudine-induced HBeAg seroconversion. *J Viral Hepat.* 2002;9(3):208-212.

82. Lee HW, Lee HJ, Hwang JS, et al. Lamivudine maintenance beyond one year after HBeAg seroconversion is a major factor for sustained virologic response in HBeAg-positive chronic hepatitis B. *Hepatology.* 2010;51(2):415-421.

83. Lee IC, Sun CK, Su CW, et al. Durability of nucleos(t)ide analogues treatment in patients with chronic hepatitis B. *Medicine (Baltimore).* 2015;94(32):e1341.

84. Lee HA, Seo YS, Park SW, et al. Hepatitis B surface antigen titer is a good indicator of durable viral response after entecavir off-treatment for chronic hepatitis B. *Clin Mol Hepatol.* 2016;22(3):382-389.

85. Lens S, Burton A, Davies J, et al. Differential changes in global and antigen-specific B cell frequencies and function associate with the outcome of HBV nucleos(t)ide analog treatment withdrawal. *J Hepatol.* 2022;77:S53-S54.

86. Li T, Liang Y, Zhang M, et al. Nucleoside/nucleotide analog consolidation therapy in hepatitis B e-antigen positive chronic hepatitis B patients: Three years should be preferred. *Hepatol Res.* 2021;51(6):633-640.

87. Liang Y, Jiang J, Su M, et al. Predictors of relapse in chronic hepatitis B after discontinuation of anti-viral therapy. *Aliment Pharmacol Ther.* 2011;34(3):344-352.

88. Lin CC, Bair MJ, Chen CJ, et al. Off-treatment efficacy of 3-year nucleos(t)ide analogues in chronic hepatitis B patients. *Kaohsiung J Med Sci.* 2016;32(1):10-15.

89. Liu F, Liu ZR, Li T, et al. Varying 10-year off-treatment responses to nucleos(t)ide analogues in patients with chronic hepatitis B according to their pretreatment hepatitis B e antigen status. *J Dig Dis.* 2018;19(9):561-571.

90. Liu YC, Jeng WJ, Liaw YF. Earlier and more severe off-tenofovir hepatitis flares than off-entecavir flares in hepatitis be antigen-negative patients. The Liver Meeting Digital Experience™. AASLD 2020.

91. Liu C, Jeng WJ, Chien RN, Liaw YF. Frequent and later off-therapy clinical relapse in hepatitis be antigen-negative patients with higher HBV surface antigen at end-of-treatment. *Hepatology.* 2021;74:15A-16A.

92. Liu YC, Jeng WJ, Peng CW, et al. Early off-therapy hepatitis flare is more severe than late flare in hepatitis be antigen-negative patients. *Hepatology.* 2021;74:453A-454A.

93. Ma TL, Hu TH, Hung CH, Wang JH, Lu SN, Chen CH. Incidence and predictors of retreatment in chronic hepatitis B patients after discontinuation of entecavir or tenofovir treatment. *PLoS One.* 2019;14(10):e0222221.

94. Manolakopoulos S, Kranidioti H, Kourikou A, et al. Long-term clinical outcome of HBeAg-negative chronic hepatitis B patients who discontinued nucleos(t)ide analogues. *Liver Int.* 2021;41(1):48-57.

95. Nagata N, Kagawa T, Hirose S, et al. Off-treatment durability of antiviral response to nucleoside analogues in patients with chronic hepatitis B. *BMC Gastroenterol.* 2016;16:38.

96. Ohlendorf V, Wübbolding M, Zu Siederdissen CH, et al. Limited value of HBV-RNA levels for the prediction of relapse after discontinuation of nucleos(t)ide analogue therapy in HBe antigen negative chronic hepatitis B patients. *J Hepatol.* 2022;77:S248-S249.

97. Paik YH, Kim JK, Kim DY, et al. Clinical efficacy of a 24-months course of lamivudine therapy in patients with HBeAg negative chronic hepatitis B: a long-term prospective study. *J Korean Med Sci.* 2010;25(6):882-887.

98. Pan HY, Pan HY, Chen L, et al. Ten-year follow-up of hepatitis B relapse after cessation of lamivudine or telbivudine treatment in chronic hepatitis B patients. *Clin Microbiol Infect.* 2015;21(12):1123.e1121-1129.

99. Papatheodoridi M, Zachou K, Hadziyannis E, et al. Kinetics of serum HBsAg and interferon inducible protein-10 (IP10) levels in non-cirrhotic HBeAg-negative chronic hepatitis B patients who discontinue entecavir (ETV) or tenofovir (TDF) therapy: results from the prospective DARING-B study. *Hepatology.* 2017;66:487A-488A.

100. Papatheodoridi M, Hadziyannis E, Berby F, et al. Predictability of serum HBcrAg, HBsAg and interferon inducible protein 10 (IP10) levels in non-cirrhotic HBeAg-negative chronic hepatitis B patients who discontinue entecavir (ETV) or tenofovir (TDF) therapy: Results from the prospective DARING-B study. *Hepatology.* 2018;68:243A-243A.

101. Park CH, Kim HY, Lee SW, et al. On-treatment and off-treatment efficacy of entecavir in a real-life cohort of chronic hepatitis B patients. *Eur J Gastroenterol Hepatol.* 2016;28(10):1179-1187.

102. Patwardhan VR, Sengupta N, Bonder A, Lau D, Afdhal NH. Treatment cessation in noncirrhotic, e-antigen negative chronic hepatitis B is safe and effective following prolonged anti-viral suppression with nucleosides/nucleotides. *Aliment Pharmacol Ther.* 2014;40(7):804-810.

103. Peng J, Cao J, Yu T, et al. Predictors of sustained virologic response after discontinuation of nucleos(t)ide analog treatment for chronic hepatitis B. *Saudi J Gastroenterol.* 2015;21(4):245-253.

104. Peng CW, Jeng WJ, Chien RN, et al. Predictors for off-therapy HBeAg sero-reversion hepatitis and HBeAg-negative hepatitis in HBeAg-positive patients discontinue NUC therapy are different. *Hepatology.* 2021;74:489A-490A.

105. Peng CW, Jeng WJ, Liu YC, et al. Distinct different relapse patterns but similar HBeAg sero-reversion rate after cessation of entecavir and tenofovir in HBeAg-positive chronic hepatitis B patients. *Hepatology.* 2021;74:476A-477A.

106. Petersen J, Buggisch P, Stoehr A, et al. Stopping long-term nucleos(t)ide analogue therapy before HBsAg loss or seroconversion in HBeAg-negative CHB patients: experience from five referral centers in Germany. AASLD Abstracts. *Hepatology.* 2011;54(4):1033A-1033A.

107. Petersen J, Buggisch P, Hinrichsen H, et al. Stopping long-term nucleos(t)ide analogue therapy before HBsAg loss in HBeAg-negative CHB patients: follow-up of long-term responders. *J Hepatol.* 2013;58:S313-S314.

108. Pocurull A, Hoyas E, Rodriguez M, et al. Low levels of qHBsAg and tenofovir therapy are associated with successful treatment withdrawal in HBeAg-negative chronic hepatitis B: results from Spanish multicentric study. *J Hepatol.* 2022;77:S857-S858.

109. Qiu YW, Huang LH, Yang WL, et al. Hepatitis B surface antigen quantification at hepatitis B e antigen seroconversion predicts virological relapse after the cessation of entecavir treatment in hepatitis B e antigen-positive patients. *Int J Infect Dis.* 2016;43:43-48.

110. Ridruejo E, Marciano S, Galdame O, et al. Relapse rates in chronic hepatitis B naïve patients after discontinuation of antiviral therapy with entecavir. *J Viral Hepat.* 2014;21(8):590-596.

111. Ryu SH, Chung YH, Choi MH, et al. Long-term additional lamivudine therapy enhances durability of lamivudine-induced HBeAg loss: a prospective study. *J Hepatol.* 2003;39(4):614-619.

112. Sah FT, Keskin O, Karakaya F, et al. P-048. Clinical outcomes after cessation of potent antiviral treatment in chronic hepatitis B patients. *Turk J Gastroenterol.* 2019;30(suppl 1):S81.

113. Santos AL, Simoes G, Cardoso H, et al. Intentional discontinuation of antiviral therapy in HBeAg-negative chronic hepatitis B: high sustained response and HBsAg seroconversion. *Hepatology.* 2019;70:317A-318A.

114. Sattayalertyanyong O, Tanwanadee T, Nimanong S. Severe hepatitis flares without beneficial effects on HBsAg clearance in non-cirrhotic chronic hepatitis B HBeAg-negative treated with tenofovir or entecavir after stopping treatment: A prospective controlled study. AASLD Abstracts. 2020:S-1289.

115. Seto WK, Hui AJ, Wong VW, et al. Treatment cessation of entecavir in Asian patients with hepatitis B e antigen negative chronic hepatitis B: a multicentre prospective study. *Gut.* 2015;64(4):667-672.

116. Seto WK, Cheung KS, Wong DK, et al. Hepatitis B surface antigen seroclearance during nucleoside analogue therapy: surface antigen kinetics, outcomes, and durability. *J Gastroenterol.* 2016;51(5):487-495.

117. Seto WK, Liu KS, Mak LY, et al. Role of serum HBV RNA and hepatitis B surface antigen levels in identifying Asian patients with chronic hepatitis B suitable for entecavir cessation. *Gut.* 2021;70(4):775-783.

118. Shin JW, Park NH, Park JH, et al. Efficacy of lamivudine re-treatment for relapsed patients after an initial lamivudine therapy in HBeAg-positive chronic hepatitis B. *J Viral Hepat.* 2005;12(4):393-397.

119. Sohn HR, Min BY, Song JC, et al. Off-treatment virologic relapse and outcomes of re-treatment in chronic hepatitis B patients who achieved complete viral suppression with oral nucleos(t)ide analogs. *BMC Infect Dis.* 2014;14:439.

120. Song MJ, Song DS, Kim HY, et al. Durability of viral response after off-treatment in HBeAg positive chronic hepatitis B. *World J Gastroenterol.* 2012;18(43):6277-6283.

121. Song DS, Chang UI, Yang JM, et al. Usefulness of stopping rule and clinical predictors for relapse after cessation of nucleoside analogues in chronic hepatitis B patients. *Hepatol Int.* 2018;12(1):S337-S338.

122. Song DS, Jang JW, Yoo SH, et al. Improving the prediction of relapse after nucleos(t)ide analogue discontinuation in patients with chronic hepatitis B. *Clin Infect Dis.* 2021;73(4):e892-e903.

123. Sonneveld MJ, Chiu SM, Park JY, et al. Probability of HBsAg loss after nucleo(s)tide analogue withdrawal depends on HBV genotype and viral antigen levels. *J Hepatol.* 2022;76(5):1042-1050.

124. Sonneveld M, Chiu SM, Park JY, et al. Predictors of HBsAg loss after cessation of nucleos(t)ide analogue therapy in Asian patients with low HBsAg levels. Poster Presentation (SAT344). In: The International Liver Congress. *J Hepatol.* 2022.

125. Su TH, Yang HC, Tseng TC, et al. Distinct relapse rates and risk predictors after discontinuing tenofovir and entecavir therapy. *J Infect Dis.* 2018;217(8):1193-1201.

126. Su CW, Wu CY, Lin JT, Ho HJ, Wu JC. Nucleos(t)ide analogue continuous therapy associated with reduced adverse outcomes of chronic hepatitis B. *J Chin Med Assoc.* 2020;83(2):125-133.

127. Su TH, Liao CH, Liu CJ, et al. High fib-4 index predicts liver decompensation after discontinuation of antiviral therapy in patients of chronic hepatitis B. *Hepatology.* 2021;74:484A-485A.

128. Suh SJ, Yeon JE, Yoon EL, et al. 486 Quantification of hepatitis B surface antigen as a predictor of off-treatment sustained virological response in chronic hepatitis B patients treated with oral nucleos(t)ide analogue. *J Hepatol.* 2012;56:S191.

129. Tseng CH, Hsu YC, Chang CY, et al. Quantification of serum hepatitis B core antibody to predict off-entecavir relapse in patients with chronic hepatitis B. *J Formos Med Assoc.* 2018;117(10):915-921.

130. Tseng TN, Hu TH, Wang JH, et al. Incidence and factors associated with HBV relapse after cessation of entecavir or tenofovir in patients with HBsAg below 100 IU/mL. *Clin Gastroenterol Hepatol.* 2020;18(12):2803-2812.e2802.

131. Tseng TN, Kuo YH, Hu TH, et al. Kinetics in HBsAg after stopping entecavir or tenofovir in patients with virological relapse but not clinical relapse. *Viruses.* 2022;14(6).

132. Tsuge M, Murakami E, Imamura M, et al. Serum HBV RNA and HBeAg are useful markers for the safe discontinuation of nucleotide analogue treatments in chronic hepatitis B patients. *J Gastroenterol.* 2013;48(10):1188-1204.

133. Tuefferd M, Crabbe M, Hu TH, et al. Novel markers to predict virological and clinical relapse onset following antiviral treatment discontinuation in chronic hepatitis B patients. *J Hepatol.* 2020;73:S139.

134. Tzu-Ning T, Chen CH, Hu TH, et al. HBsAg changes after cessation of entecavir or tenofovir in HBeAg-negative patients who experienced virological relapse without clinical relapse. *J Gastroenterol Hepatol.* 2021;36:62-63.

135. Wang L, Liu F, Liu YD, et al. Stringent cessation criterion results in better durability of lamivudine treatment: a prospective clinical study in hepatitis B e antigen-positive chronic hepatitis B patients. *J Viral Hepat.* 2010;17(4):298-304.

136. Wang Y, Huang G, Xia J, et al. A real-life study (RWS) of long-term outcomes after discontinuation of NUC monotherapy, PEG IFN-α monotherapy and their combo-therapy in patients with HBeAg-positive chronic hepatitis B (CHB). *Hepatol Int.* 2020;14:S61–S62.

137. Wang FD, Zhou J, Li LQ, et al. Serum pregenomic RNA combined with hepatitis B core-related antigen helps predict the risk of virological relapse after discontinuation of nucleos(t)ide analogs in patients with chronic hepatitis B. *Front Microbiol.* 2022;13:901233.

138. Wong VW, Wong GL, Tsang SW, et al. Long-term follow-up of lamivudine treatment in patients with severe acute exacerbation of hepatitis B e antigen (HBeAg)-positive chronic hepatitis B. *Antivir Ther.* 2008;13(4):571-579.

139. Xie Y, Li M, Ou X, et al. HBeAg-positive patients with HBsAg< 135IU/mL or HBcrAg< 3.6 log U/mL have more chance to be HBsAg loss after nucleos(t)ide analogue cessation. *J Hepatol.* 2022;77:S278-S279.

140. Xu WX, Li YM, Li JG, et al. The 96-week clinical outcomes after cessation of nucleos(t)ide analog treatment in chronic hepatitis B patients. *Gastroenterol Rep (Oxf).* 2021;9(4):313-322.

141. Xu W, Shu Zhu S, Yeqiong Zhang Y, et al. Hepatitis B surface antigen kinetics after nucleos(t)ide analogues cessation and in subsequent retreatment in noncirrhotic chronic hepatitis B patients. APASL 2022. 16(S1):S177-S178.

142. Yao CC, Hung CH, Hu TH, et al. Incidence and predictors of HBV relapse after cessation of nucleoside analogues in HBeAg-negative patients with HBsAg ≤ 200 IU/mL. *Sci Rep.* 2017;7(1):1839.

143. Yen CL, Su WW, Wu CS, et al. Virological and clinical outcomes after cessation of nucleos(t)ide analogue therapy for chronic hepatitis B-A prospective cohort study in Central Taiwan. *Gut.* 2018;67(2):A105.

144. Yoon SK, Jang JW, Kim CW, et al. Long-term results of lamivudine monotherapy in Korean patients with HBeAg-positive chronic hepatitis B: response and relapse rates, and factors related to durability of HBeAg seroconversion. *Intervirology.* 2005;48(6):341-349.

145. Zhang M, Liu F, Liu Z, et al. Impact of varied consolidation durations on sustained virologic response to nucleos(t)ide analogues in HBeAg-positive CHB patients: A propensity score matching analysis. *Hepatol Int.* 2017;11(1):S740.
